# Global economic trade-offs between wild nature and tropical agriculture

**DOI:** 10.1371/journal.pbio.2001657

**Published:** 2017-07-21

**Authors:** Luis R. Carrasco, Edward L. Webb, William S. Symes, Lian P. Koh, Navjot S. Sodhi

**Affiliations:** 1 Department of Biological Sciences, National University of Singapore, Republic of Singapore; 2 Environment Institute, School of Biological Sciences, The University of Adelaide, Adelaide, South Australia, Australia; Princeton University, United States of America

## Abstract

Global demands for agricultural and forestry products provide economic incentives for deforestation across the tropics. Much of this deforestation occurs with a lack of information on the spatial distribution of benefits and costs of deforestation. To inform global sustainable land-use policies, we combine geographic information systems (GIS) with a meta-analysis of ecosystem services (ES) studies to perform a spatially explicit analysis of the trade-offs between agricultural benefits, carbon emissions, and losses of multiple ecosystem services because of tropical deforestation from 2000 to 2012. Even though the value of ecosystem services presents large inherent uncertainties, we find a pattern supporting the argument that the externalities of destroying tropical forests are greater than the current direct economic benefits derived from agriculture in all cases bar one: when yield and rent potentials of high-value crops could be realized in the future. Our analysis identifies the Atlantic Forest, areas around the Gulf of Guinea, and Thailand as areas where agricultural conversion appears economically efficient, indicating a major impediment to the long-term financial sustainability of Reducing Emissions from Deforestation and forest Degradation (REDD+) schemes in those countries. By contrast, Latin America, insular Southeast Asia, and Madagascar present areas with low agricultural rents (ARs) and high values in carbon stocks and ES, suggesting that they are economically viable conservation targets. Our study helps identify optimal areas for conservation and agriculture together with their associated uncertainties, which could enhance the efficiency and sustainability of pantropical land-use policies and help direct future research efforts.

## Author summary

Tropical forests are often destroyed to clear land for agriculture or to harvest forestry products, such as timber. However, the benefits derived from agriculture and these products are countered by the costs to the environment and the loss of ecosystem systems (the benefits that nature provides to humans). Little is known about how the economic benefits and costs of deforestation vary on a global scale. Knowing the distribution of benefits and costs would help identify regions where deforestation is most and least beneficial and thus could help select areas to focus conservation efforts. We studied the trade-offs between agricultural benefits, carbon emissions, and losses of multiple ecosystem services (ES) in tropical deforested areas around the world. We find large differences between costs and benefits globally. For instance, we identify the Atlantic Forest, areas around the Gulf of Guinea, and Thailand as areas where the benefits from agricultural conversion are greater than environmental costs, which could make it difficult to incentivize and implement biodiversity conservation strategies that are based on payments to farmers. By contrast, Latin America, insular Southeast Asia, and Madagascar represent areas with low agricultural benefits and high environmental costs. This suggests that these regions are economically viable conservation targets. Our study helps identify strategies to enhance the sustainability of land-use policies in the tropics.

## Introduction

Growing global demands for food and biofuels generate pressures for deforestation across the tropics [1]. Much of this deforestation is carried out without information on the spatial distribution of benefits and costs of deforestation [2]. Studies estimating the trade-offs between the economic value of multiple forest ecosystem services (ES) and agricultural conversion have been largely constrained to local and national case studies [3–7]. On the other hand, increasingly detailed, spatially explicit analyses of the global trade-offs between biodiversity and carbon emissions [8–12] and between carbon emissions and agricultural production [13,14] have been produced. Analyses that combine both approaches to analyse the economic trade-offs between agriculture and multiple tropical forests’ ES at the global level are, however, lacking. Identifying the spatial distribution of trade-offs between economic net losses and gains resulting from deforestation is important as it can help identify optimal areas for conservation and agriculture, thus informing pantropical land-use policies.

Availability of spatial datasets on tropical deforestation [15], agricultural crop distributions [16], and potential yields [17] and economic values of ES in tropical forests [18] presents a unique opportunity to comprehensively evaluate the contemporary and future economic trade-offs and inefficiencies between carbon emissions, multiple ES, and agricultural conversion linked to tropical deforestation.

Here we present a spatially explicit analysis using deforestation and crop distribution data for the period of 2000–2012 [15]. We compared agricultural benefits to both foregone avoided carbon emissions values and lost ES by developing a spatially explicit meta-analysis of the total economic value of ES in tropical forests (Materials and methods). By overlaying the estimates of carbon emissions and ES values onto spatial analyses of land-use change and agricultural rents (ARs), we quantified the net economic trade-offs and inefficiencies associated with tropical deforestation. We accounted for uncertainty using bootstrapping, Monte Carlo simulation methods, and different scenarios (Materials and methods). Scenario A presents net agricultural rents of national crops (i.e., assuming that crops replacing forests can stochastically be those already existing within the country and deducting production costs); scenario C presents net agricultural rents of the economically highest potential crop for that cell (among the top 10 crops in terms of area and production value in the tropics when considering their potential yields, observed prices, and costs and ignoring know-how and cultural and infrastructure limitations; see Materials and methods), deducting production costs; and scenarios B and D are respectively similar to A and C except that production costs are not deducted. Scenarios A and B are designed to represent the contemporary deforestation scenario that makes an attempt to capture a more realistic agricultural expansion (see Materials and methods for an evaluation of the plausibility of these scenarios). Scenarios C and D are designed to emulate the hypothetical conversion of land into the highest-rent crops in the long term as a response to increasing global agricultural demand.

## Results

**Fig 1:**
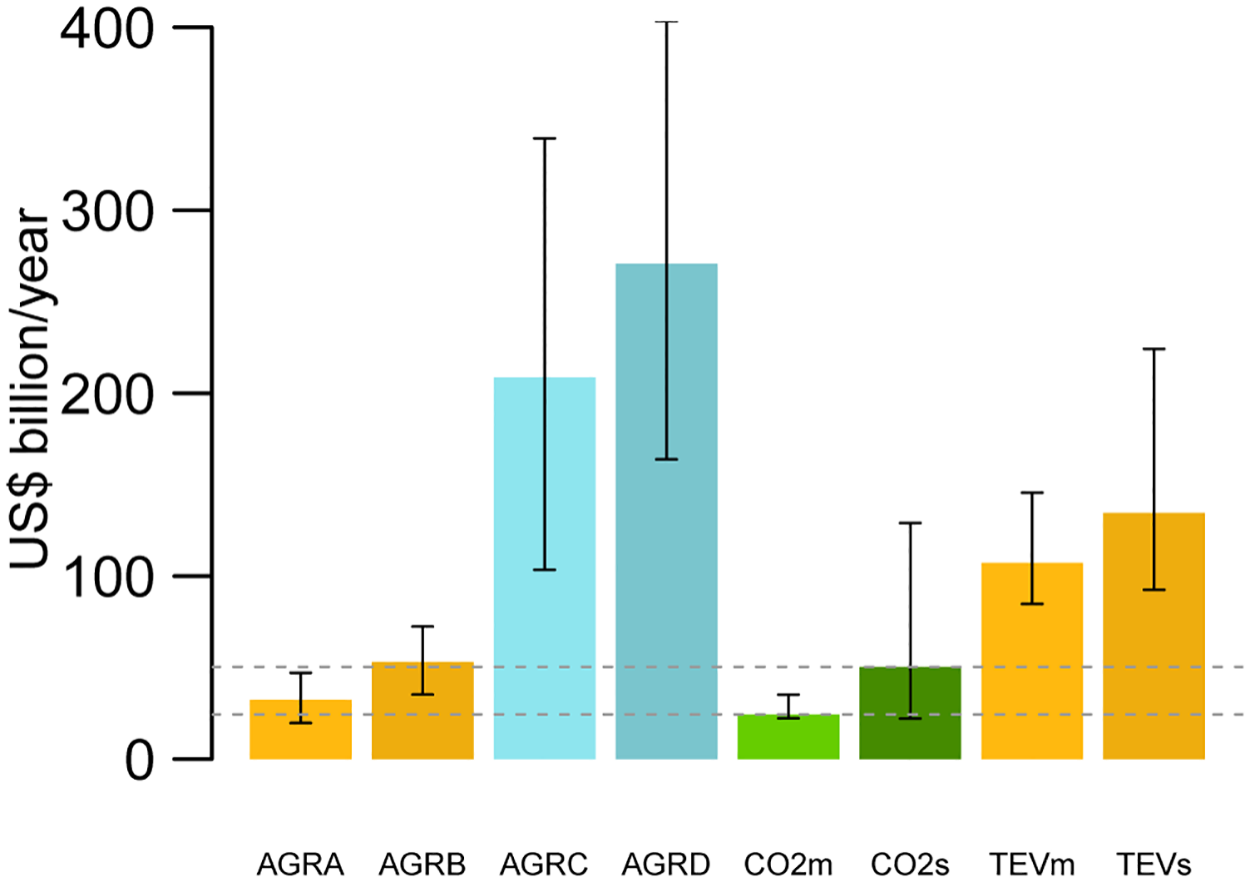
Comparison (2000–2012) of the annual agricultural value gained on deforested land versus externalities consisting of carbon emission and lost ecosystem services (ES) value. AGRA, AGRB, AGRC, AGRD: median agricultural rents generated under scenarios A, B, C, and D respectively. CO_2_m and CO_2_s: median value of carbon emissions at market and social price levels, respectively. TEV: total ecosystem service value. TEVm and TEVs: median value of TEV including carbon emissions at market and social price levels, respectively. Error bars indicate the 2.5th and 97.5th percentiles of the uncertainty distribution of outcomes. Dotted horizontal lines denote the median of carbon emissions externalities under market and social prices.

**Fig 2:**
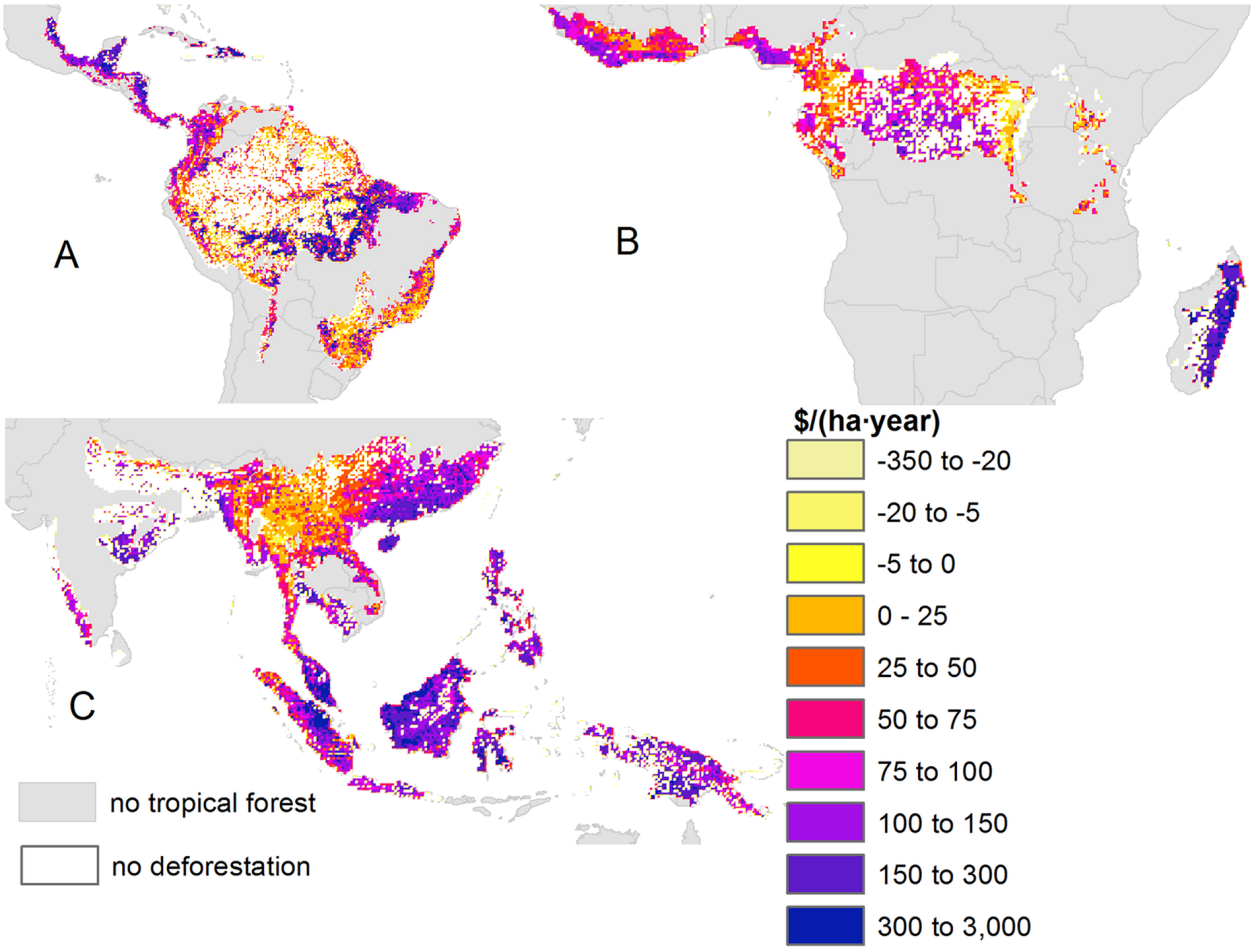
The economic implications of deforestation in tropical forests from 2000 to 2012. Comparison of carbon emissions assessed at market prices plus loss of ecosystem services (ES) values minus gains of agricultural rents. Replacement of land by those crops in the tropical forest biome already present in the country is assumed (Scenario A). The median values of the simulations are shown. Maps corresponding to the 2.5th and 97.5th percentiles of the uncertainty distribution of outcomes are available in S3 and S4 Figs.

Our meta-analysis of ES identified 3 top models with high support (S1 Table and S1 Fig in Supporting information). The results pointed towards the influence of valuation method and type of ES (although not statistically significant in the case of type of ES) on the value of ES. We found a positive relationship of value with temperature and negative with year of publication and bird species richness (S1 Fig). These models presented an improvement of predictive accuracy of 43%–49% with regards to direct benefits transfer at the global and regional levels (S1 Table) and predictive versus observed regression slopes of 0.879–0.884 (S2 Fig). The dataset was also representative of the tropical forest biome (S2 Table). The predictions from the ES meta-analytic model presented, however, a wide range of uncertainty (Figs 1 and 2; its uncertainty is described in S3 and S4 Figs).

Although the results are spatially explicit and present large levels of heterogeneity across space (Fig 2), to help dissect the findings, we present first results aggregated at the global, regional, and national scales before describing specific spatial heterogeneities. Under scenarios A and B in which a forest is replaced with crops already present in the country, the global annual net and gross agricultural benefits were on average I$32,000,000,000 (95% uncertainty range [UR] = I$19–I$47,000,000,000/year, Scenario A) and I$53,000,000,000 (UR = I$35–I$72,000,000,000/year, Scenario B). Under the scenarios in which a forest was replaced by crops with the highest potential rents, the net and gross annual values increased substantially to I$209,000,000,000/year (UR = I$103–I$339,000,000,000/year, Scenario B) and I$271,000,000,000 (UR = I$164–I$403,000,000,000/year, Fig 1).

Annual externalities only related to carbon emissions were on average I$24,000,000,000 (UR = I$22–I$35,000,000,000/year) at market prices and I$50,000,000,000 (UR = I$22–I$129,000,000,000/year) at social prices (Fig 1, S5 and S6 Figs, Materials and methods). However, when total ES value (TEV) (that includes the value of carbon emissions) is considered, annual externalities increased to I$107,000,000,000 (UR = I$85–I$146,000,000,000/year at market prices) and I$135,000,000,000 (UR = I$93–I$224,000,000,000/year at social prices). Thus, at the aggregate (global) level, agricultural expansion under scenarios A and B provided on average a net benefit only when compared with the value of carbon at market prices. In comparison with all other ES valuations, agricultural expansion under scenarios A and B resulted on average in net losses (although the uncertainty ranges of scenarios A and B overlapped with the lower uncertainty bound of carbon emissions under social prices). Under a hypothetical future scenario in which all crops that replaced deforestation presented maximum rents, median annual agricultural rents exceeded on average the value of externalities because of carbon emissions, regardless of pricing, as well as TEV (Fig 1).

**Fig 3:**
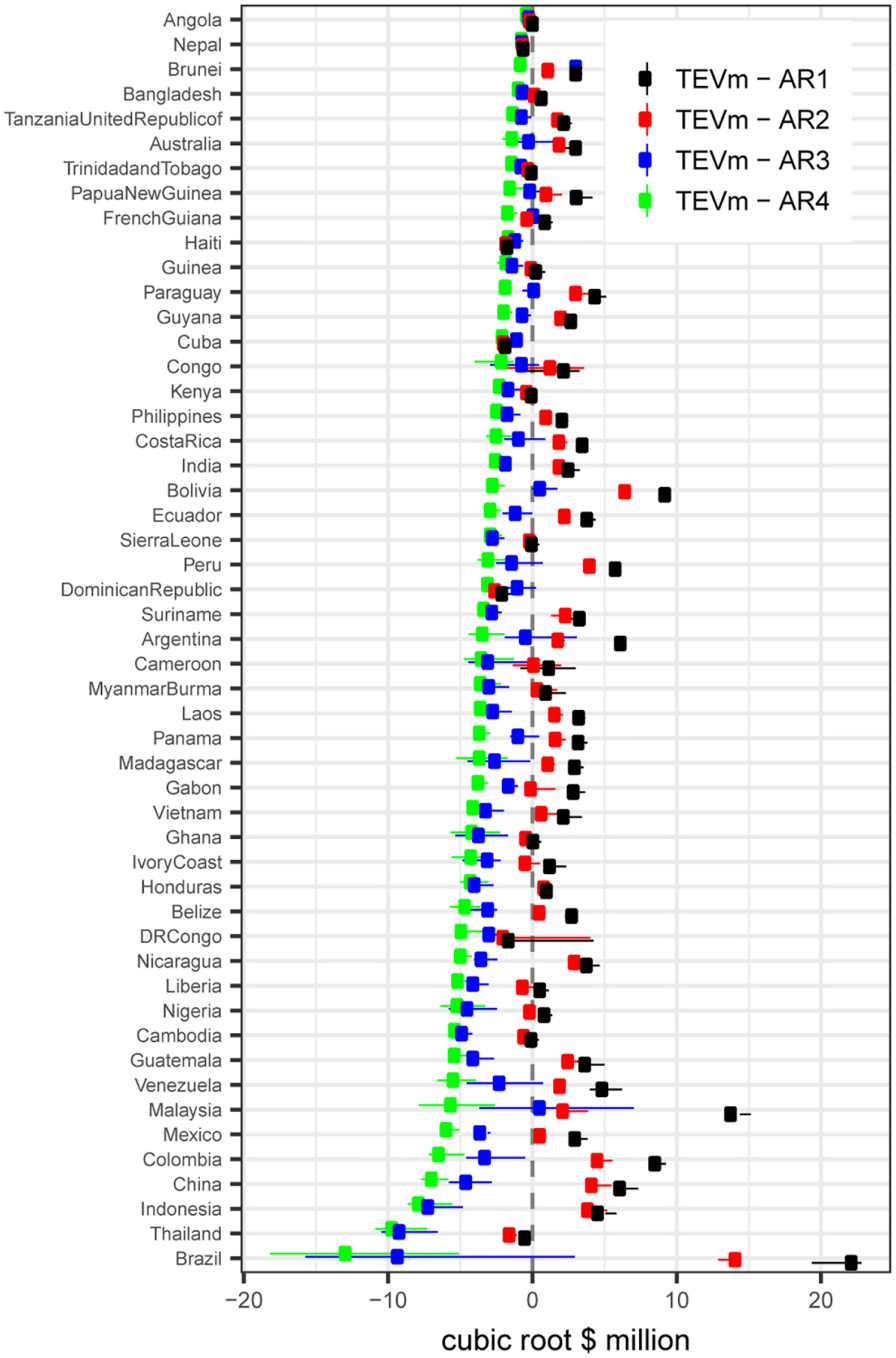
Annual per-country net benefits of converting tropical forests to agriculture for the years 2000–2012 compared to total ecosystem services value (TEV) losses. This figure shows the result of deducting cubic root–transformed agricultural rents (ARs) of the crops replacing forests under 4 different scenarios (A, B, C, and D with corresponding agricultural rents AR1, AR2, AR3, and AR4) to the cubic root TEV based on the market price of carbon (TEVm)—i.e., TEV^1/3^ –AR^1/3^. Error bars indicate the 2.5th and 97.5th percentiles of the uncertainty distribution of outcomes.

The distribution of trade-offs varied substantially between countries (Fig 3, S7, S8 and S9 Figs).

Countries like Brazil, Indonesia, Peru, and Nicaragua presented high contemporary net losses under scenarios A and B but the capacity to obtain net gains if maximum rent crops were realized in scenarios C and D, pointing towards low production costs relative to further gains from intensive cash crops such as soybean, oil palm, maize, and sugar cane (Fig 3, S7, S8 and S9 Figs). In contrast to most countries, several countries presented large differences between scenarios C and D, suggesting high production costs that would hinder intensive cash crops expansion. In this group were countries like Malaysia, Panama, Argentina, Paraguay, and Australia (Fig 3, S7, S8 and S9 Figs).

When considering the spatial distribution of losses under scenario A, a large heterogeneity was present within and between countries, showing where agricultural conversion was economically efficient and areas where it was inefficient (Fig 2, S3 and S4 Figs).

Net losses of total ES occurred on average in most of South America (except the Atlantic Forest), Madagascar, the Philippines, centre and northern parts of Borneo, Papua, and Indochina, excluding parts of Thailand and Malaysia. Conversely, net gains occurred in western tropical Africa, around the Gulf of Guinea and the eastern part of the Congo Basin, Thailand, parts of Sumatra, the Atlantic Forest, and areas in Bolivia and Peru (Fig 2, S3 and S4 Figs show the large heterogeneity of these results across space). The areas with large ES losses (Fig 2) were largely in agreement with areas where carbon emissions out-competed agriculture when considered alone under social prices, with the exception of areas such as Southeast Asia and Madagascar, which presented also net losses (S10 and S11 Figs). The maps of net gains from agricultural conversion presented large uncertainty towards higher ES values: the 2.5th percentile map resembled the 50th percentile map, but the 97.5th percentile map showed a majority of net losses from agricultural conversion under scenarios A and B (Fig 2, S3, S4, S12, S13 and S14 Figs). By contrast, under scenario C, most agricultural conversion presented net gains under the 2.5th and 50th percentile maps (S15 and S16 Figs) except areas such as Latin America, Congo Basin, Papua and north of Borneo under the 97.5th percentile map (S17 Fig), highlighting the difficulty to meet future agricultural opportunity costs in extensive areas using ES and carbon emissions alone.

Our analyses to evaluate the plausibility of the scenarios considered showed that scenarios A and B are plausible given the spatial contagion of crop expansion illustrated by oil palm expansion in Southeast Asia (S18 Fig; relationship between distance from existing plantation and new oil palm conversion β = –0.06, *p*-value < 0.01) and that we could only find 24 new crop–country combinations (out of the 2,903 considered) with either missing area information or 0 area reported during the period of study (S3 Table).

## Discussion

Our analysis reveals large spatial heterogeneity in net losses or gains from the agricultural conversion of tropical forests across subnational to global scales.

Deforestation in Latin America (except the Atlantic Forest) was in general identified to generate net losses because of low agricultural rents—i.e., if carbon emissions and ES values were internalized in the planning of individuals and corporations, these are not viable regions for conversion to agriculture under present conditions—and relatively high carbon emissions and ES values. These results were largely robust to uncertainty under scenario A (Fig 2, S3 and S4 Figs). By contrast, Southeast Asia (mostly north of Sumatra, Thailand and the Malayan Peninsula) were identified to generate net agricultural gains and to be a less preferred target for conservation interventions such as REDD+ investments. However, these results were susceptible to uncertainty (net losses of ES because of agricultural conservation occur as well for the 97.5th percentile map, S4 Fig). The identification of these regions agrees with previous studies evaluating the optimal allocation of REDD+ funds [8]. Our results also agree with global trade-off analyses indicating the high agricultural yields in Southeast Asia versus low yields in the Neotropics and Afrotropics [13], corroborating a crop advantage over carbon in Southeast Asia [14]. In general, our results show good agreement with the identification of areas where agriculture generates net gains in Thailand, the border of India and Nepal, and the Malayan Peninsula [14]. Our analyses, by incorporating the economic dimension beyond yields alone, further suggests that agricultural rents in Southeast Asia are able to surpass the value of carbon emissions, suggesting that high agricultural rents and thus conservation opportunity costs could compromise the viability of REDD+ projects in SE Asia [19], although, yet again, these results are subject to uncertainty in the upper-level value of ES.

Our analyses do not include trade-offs with biodiversity. Factoring biodiversity may mean, however, that conservation funds would need to be invested in Southeast Asia [8,9], which hosts 4 biodiversity hotspots and combines high levels of endemism and threat. This highlights a strong trade-off between agricultural rents and ES with biodiversity in Sumatra and the Malayan Peninsula. This corroborates also the ES–biodiversity negative trade-off found by the ES meta-analytic models: high economic value of ES requires high density of beneficiaries, while high levels of biodiversity require, by contrast, low levels of disturbance and hence less beneficiaries [20]. This trade-off, although pervasive, is not necessarily dominating across all the tropics, as it can still be modulated by agricultural opportunity costs. For instance, because of low agricultural rents, and with high robustness to uncertainty under scenario A, we identified highly biodiverse areas with high potential for species extinctions such as the Philippines, Borneo and Madagascar as net losers from agricultural conversion, showing them as economically viable conservation targets.

We identified 2 different dynamic interpretations of the trade-offs between agriculture and forests depending on whether contemporary and hypothetical maximum rent crop conversion were considered. Under contemporary conversion, deforestation produced net negative global externalities from 2000–2012 when the crops present in each country replaced forests, a realistic scenario given that existing local crops reflect cultural and labour constraints (e.g., labour constraints make the adoption of oil palm in countries like Brazil unlikely), and there is high spatial autocorrelation inherent to agricultural land use [21] (S17 Fig). Our analysis of time series of crop areas further confirms the plausibility of scenarios A and B. These net losses are likely to be even more negative than we report because the analyses assumed that all conversion resulted in immediate and sustained productive agriculture and did not incorporate estimates of degraded land resulting from agricultural abandonment, such as *Imperata* grasslands in Southeast Asia [22]. On the other hand, crops at the contemporary deforestation frontiers are expected to evolve towards higher-rent crops with better yields and economies of scale. Under these intensification scenarios in which the highest rent crop is adopted, agricultural rents surpass the externalities of carbon emissions and combined ES with very few exceptions. This points towards pantropical runaway costs for conservation [23] that will be very difficult to match under current carbon prices in the long term. It should be noted, however, that the value of ES can also change in space and time [24] and would be expected to increase as tropical forests become more scarce [25]. Similarly, the social price of CO_2_ increases through time as the impacts of climate change unfold and the gross domestic product (GDP) increases [26]. Our current analysis is, however, done using the CO_2_ prices from the period of study. Future research would thus need to update the changing value in ES, CO_2_, and agricultural rents.

Our analyses have several limitations. We compounded several datasets and analyses that required uncertainty propagation through modelling. Although we dealt with this problem using bootstrapping and Monte Carlo simulation methods, a research priority would be to reduce data paucity on ES valuation studies (so that individual models for each ES can be created) and the location of crops replacing forests, which would substantially reduce the uncertainty attached to our results. Although the cross-validation analysis and comparison with direct benefit transfer suggest that our ES meta-analysis is a step forward compared to direct benefit transfer and assumptions of constant ES values per biome across space, the results related to the ES meta-analysis should be contemplated while bearing in mind the limitations associated to the dataset. Although the uncertainty in the predictions of the ES models was considered, the individual studies from The Economics of Ecosystems and Biodiversity (TEEB) dataset that were used to develop the meta-analytic models present themselves inherent inaccuracies and uncertainties that cannot be captured by bootstrapping or cross-validation of the ES models. Estimating the value of ES is very challenging as value depends, in a complex manner on the social, ecological, and economic context of the location (e.g., flood protection values will depend on the hydrological characteristics of the catchment and the communities that can be affected by the flood). Although we attempted to capture the contextual realities as much as possible statistically, there is no guarantee that all the variables influencing value were included in the meta-analytic models or that the variables we used had sufficient resolution to capture the nuanced socioecological dynamics that shape values in each specific location. As such, we highlight that there is potential for wide error in the values of ES used for analysis and that these errors may have escaped our treatment of uncertainty. In this respect, the trade-off maps between CO_2_ and agricultural rents present higher confidence than those between TEV and agricultural rents, making it essential that future work strives at generating and synthetizing further ES valuation studies. Because of data paucity, we did not estimate net externalities from agriculture, which is conservative given that agricultural activities tend to generate negative externalities (e.g., water pollution, increased flooding, and greenhouse emissions) that are greater in magnitude than the positive externalities (landscape aesthetic values, waste sink) [27]. Our analyses were also static and did not consider market feedbacks through international trade. Market feedbacks can modify agricultural rents in the region where land use occurs and can indirectly modify distant lands through land displacement [28]. For instance, drastic intensification or expansion of oil palm would lead to increases in supply that could cause prices to drop, presumably preventing further oil palm expansion elsewhere [29,30]. Further research is needed to understand the role of global market feedbacks on conservation [31]. Another inherent trade-off with the global scale of our analyses is that our results are aggregate in nature and fail to incorporate the necessities of the actual actors behind deforestation. Our maps are thus only intended to support global-scale analyses and should not be used for local decision-making. Our analyses would need to be complemented with on-the-ground, context-dependent studies to evaluate the actual distribution of winners and losers from agricultural conversion. We also note that although economic valuation of ES is fundamental to support land-use planning, valuation of ES is ultimately only 1 decision tool for policy makers. Reliance on economic valuation alone may not lead to the optimal conservation outcomes [32], and other criteria such as biodiversity and social equity would be needed to support conservation programs.

Other limitations because of data paucity were that our estimates of the carbon fraction of biomass were based on a constant conversion factor [33,34]. In reality, wood density is variable across tree species. Future work on carbon emissions estimation would benefit from combining datasets of wood density per species [35] and tree species maps that could be generated with future developments of airborne imaging spectroscopy [36]. Another limitation is that we could not find pantropical maps of peatland depths and resorted to using depth measures from peatlands in Indonesia [37] as a surrogate. The discovery of tropical peatlands and estimation of their depth is currently ongoing. For instance, one of the largest peat swamp forests (145,500 km^2^) was recently discovered in the Cuvette Centrale depression in the central Congo Basin, on the border of the Republic of the Congo with the Democratic Republic of the Congo [38]. Considering the large potential carbon emissions of its conversion would change our results in the areas of the Cuvette Centrale depression (confluence of the rivers Congo and Ubangi) corresponding to the Democratic Republic of Congo from small net losses from agricultural conversion (Fig 2) to large net losses. Our results could thus be refined when global maps of peatland depth become available.

Our results are helpful in that they help map the uncertainty associated to agriculture–ES trade-offs that can be used to motivate further research in the tropics, areas that receive comparatively much lower research efforts [39]. These results could also help identify the tropical trade-offs between carbon emissions, multiple ES, and agricultural rents. These maps can be used as a starting point to devise spatially efficient conservation and agricultural development policies aimed at internalizing the value of ES. Three main groups of policy interventions (namely regulations and community-based and economic instruments) have been implemented with varied outcomes [40]. Among regulations, the “polluter pays” principle, conditioning production subsidies on environmental performance, and interventions directed at the supply chain (e.g., removal of farmer credits was effective at slowing down deforestation in Brazil [41]) could be considered to guarantee provision of multiple ES. Among economic instruments, and besides REDD+, taxes on agricultural inputs, taxes on consumption, and agri-environmental schemes—which are widely implemented in high-income regions like the European Union—could also be considered in the tropics. Agri-environmental schemes could be considered as flexible interventions to promote activities that enhance provision of ES at the local scale [40]. As a drawback, agri-environmental schemes are costly to implement and to monitor, which may not be viable in low-income settings. In this respect, our results can help identify the largest ES value–agricultural rent differentials as areas where their potential economic viability is highest. Another limitation is that all these interventions could displace production elsewhere through indirect feedbacks and rebound effects. Land-use zoning, certification, and spatial strategic deployment of agricultural innovations could be considered to prevent rebound effects and displacement [42].

Although knowing the spatial distribution of trade-offs and their large associated uncertainty is still a long way from identifying the most effective policies to internalize ES losses into agricultural production, the developed maps can be useful to support evidence-informed spatial policies and to identify areas of high-potential agricultural rents where potential rebound effects could occur. Considering these policies is imperative; the alternative is a spatially inefficient agricultural conversion of tropical forests at the expense of the loss of valuable ES and biodiversity.

## Materials and methods

### Analyses overview and scope

We obtained spatial information on the distribution and magnitude of loss of tropical rainforests from 2000 to 2012 [15]. On these deforestation maps, we overlaid data on the distribution and of current and potential yields of major commercial crops, pastures, and livestock density [16,17,43] and developed meta-analytic models of ESs and estimates of carbon emissions. These meta-analytic models are an update of Carrasco et al. [20] in which we increased the time resolution of the explanatory variables, used information theory and switched to mixed-effects models without variance structures to allow bootstrapping of the models. Potential yields have been determined using a rain-fed land productivity and a water-balance model combined with soil moisture and temperature radiation information integrated in a crop growth model [17].

The combination of these analyses yielded a pantropical spatial map of cells for the distribution of tropical forest loss, the distribution of agricultural rents generated, ESs lost, and carbon emitted. Bootstrapping and Monte Carlo simulations were used to estimate uncertainty in these maps.

We restricted our analysis to the 51 countries where deforestation was detected from 2000 to 2005 [44] (Fig 3). The spatial extent of the analysis was the tropical forest biome in which we used a grid of 0.1° resolution, leading to 159,458 map cells analysed. These analyses were done using the R environment, and ArcGIS version 10.2.1 was used to develop all the maps.

### Economic impacts: Carbon emissions

We estimated the carbon dioxide emitted from deforestation because of losses in carbon aboveground, belowground, and stored in dead organic matter because of conversion from forests to agriculture. Given the large uncertainty on its estimates in the literature [45], carbon stored in the soil was conservatively assumed to remain the same from the transition from forest into agriculture. We did, however, consider the carbon emitted if peat soil was converted to agriculture. Geographic information systems (GIS) maps of biomass above and belowground stored in forests was obtained from Ruesch and Gibbs [46]. Biomass was expressed as carbon tonnes per hectare content using a carbon fraction of biomass [33,34]. Tables 2.2 and 2.3 in IPCC [34] were used to estimate the amount of carbon stored in soil and dead organic matter in tropical forests. Carbon in soil within peat swamps was corrected considering the distribution of peat swamps, and because of data paucity, depth was estimated from peat swamps in Indonesia [37,47]. All carbon estimates were expressed as tonnes of carbon dioxide per hectare. We employed 2 carbon price scenarios: (i) market prices (US$13.6/tC), the average of the market prices per tonne of carbon from August 2011 to October 2016 using the price of California Carbon Allowance Futures [48]; and (ii) social prices (average of US$30/tC), based on the White House estimates published in the year 2016 referring to the year 2010 using a discount rate of 3% under 5 different socioeconomic emissions scenarios of the Dynamic Integrated Climate-Economy model (DICE) [26].

### Economic losses: Total economic value of ES

#### Challenges when performing benefit transfer of ES

Creating maps of ES values is challenging because of the scarcity of ES valuation estimates. Benefit transfer methods are used to extrapolate from known studies to other locations based on similarities with spatial covariates. Benefit transfer is, however, challenging because the valuation method, presence of agents that will benefit from the service, level of supply of the service, time of analysis, and contextual variables describing the socioecological system that vary across space can influence the value of the ES and need to be controlled for [49]. GIS and statistical spatial metamodels that account for these factors can reduce these caveats [50]. One way to ascertain the reliability of the benefit transfer is to compare the distributions of the explanatory variables within the meta-analysis dataset and the entire tropical biome [51]. We thus verified that the ES dataset used was representative of the tropical forest biome (S2 Table, [20]).

#### Data collection

We considered all ESs classified by TEEB [18] but excluded supporting services to avoid double counting [52]. We selected the valuation studies from the TEEB dataset that were conducted on tropical forests—arguably the most comprehensive quality-verified ES valuation dataset. To ensure that observations were independent we only kept studies that were not based on benefit transfer. We excluded studies without a specific location (e.g., studies at the national or regional level were excluded). We also excluded studies that did not identify values for specific ESs and did not report specific valuation methods (i.e., studies reporting TEVs were not considered). We homogenized the currency to international dollars of 2016. All currencies were expressed per unit of area and year. To do that we used the area of the forest and information on discounting rates from each study to annualize values. Studies not reporting the area of forest were inputted as 0 by TEEB, and we excluded studies that reported net present values but did not provide discount rates or time horizons. This led to a total of 30 studies and 78 observations (S1 Data).

#### Variables

Variables were chosen to explain the variance because of the type of study and how environmental and socio-economic conditions changed across space. We also accounted for sources of nonindependence in the data. We employed 3 sets of predictors. The first considered the characteristics of the study such as ES type, whether the study was peer reviewed, and the year it was published. The second group included variables describing how environmental and socio-economic factors varied spatially. These included mean temperature and precipitation [53] to control for their effect on ecosystem function, accessibility (quantified as time to travel to the nearest city of at least 50,000 habitants to control for reachability to the ES of those benefitting from them) [54], number of habitants per unit of area [55], altitude [53], spatially explicit GDP [56], forest area (to control for supply level of ES), whether the location was within a protected area [57], species richness of birds, amphibians, small mammals, and vascular plants [58] that were included to control for their effect on ecosystem function, and carbon density as a surrogate for forest type [46]. We used country as a random effect to account for sources of nonindependence and variables such as corruption and sociopolitical and institutional factors distributed at the country level. For predictors such as density of habitants and spatially explicit GDP for which different maps were available across time, we chose the maps closest to the year of the study. Most of the predictors were selected based on existing theory and their capacity to influence the economic values of ES. Some variables, however, are surrogates of other variables for which there is not information. For instance, climatic and species richness predictors act as surrogates of ecosystem function. Density of carbon is a surrogate for forest type (e.g. primary versus secondary).

#### Statistical analyses

The mathematical description of the models is:

$$\text{log}(value_{\text{i}})=\alpha +\overset{J}{\underset{1}{\sum }}\beta_{j}X_{ji}+a_{c}+\varepsilon_{i}\text{ where }c_{m}\sim N\left(0,\sigma_{{}^{1}}^{2}\right)\text{; }\varepsilon_{i}\sim N\left(0,\sigma^{2}\right)$$

where *value*_*i*_ is the dependent variable, the observation of the ES value *i*; *i* corresponds to each observation in the dataset (i.e., an estimation of the economic value of a specific ES in a specific location [the dataset is available in S1 Data]); *α* is the intercept; *β*_*j*_ is the regression coefficient for the *J* predictors describing the type of study, environmental and socioeconomic context (*X*_*ji*_); *a*_*c*_ is the random intercept for country *c*. *a*_*c*_ is assumed to follow a normal distribution with mean 0 and variance *σ*_*1*_^*2*^; and *ε* is the error term.

To evaluate collinearity, we initially fitted a linear regression with the main effects of the variables. We used variance inflation factors and evaluated departures from the assumption of homoscedasticity by visually evaluating graphs of the residuals versus fitted values and graphs of residuals versus each of the predictors and by using a Breusch–Pagan test from the library car in R on an equivalent linear model with country as a fixed effect. The model presented multicollinearity as a result of the small number of observations per ES type and valuation method [20]. To solve this we clustered these two variables in groups. ES types were now coded as cultural, provisioning, and regulating and valuation method was coded as cost-based, stated preference, and revealed preference [20]. We assessed whether there were problems of spatial autocorrelation using semivariograms of model residuals. We also tried to model spatial autocorrelation using the mean distance between observations as a random slope [60], but this did not improve the fit. S19 Fig shows the semivariograms of models’ residuals where no problems of spatial autocorrelation were observed; AIC of the models attempting and not attempting to account for spatial autocorrelation through the random slope were 431 and 429, respectively.

Because multiple alternative mechanisms could be at play in the determination of ES value, we used an information theoretic approach considering 256 models [61] with the dredge function in the R package MuMIn [62]. The proposed models were constructed using the predictors as main effects and country as random intercept. The models were fitted using the package lme4 [63]. After the models were fitted, we performed model selection based on the AIC corrected for small sample size (AICc). We used 2 AICc units of difference with the best model as a cut-off for models with high support [61]. The models that met that cut-off were averaged together and further assessed with regards to the assumptions of homoscedasticity and normality in the distribution of the residuals. We did not observe problems of heteroscedasticity (Breusch–Pagan test *p*-values of 0.17, 0.25, and 0.29 in the top supported models) and nonnormality.

#### Model predictions

We carried out 3 sets of predictions in each cell in the map for the 3 valuation types and then averaged the predictions. We used GIS to obtain information from each predictor in each location of the map. Some predictor variables in the dataset did not have equivalent spatial information (year of publication and area of forest). In these cases we kept them fixed in their mean values while making predictions. Only the fixed-effects part of the model was used to make predictions in countries for which a random effect could not be estimated from the dataset. The predictions for each type of ES for each cell where then aggregated into: 5 types of provisioning services, 7 types of regulating services, and 5 types of cultural services from the TEEB dataset while excluding 1 regulating service to avoid double counting with the estimates of carbon emissions. Each of the three selected models was used to make predictions for each cell and these were combined as a weighted average using the weights of each model (S1 Table).

#### Model cross-validation and predictive accuracy

Models fitted to small datasets risk being dominated by single observations or overfitted, making them unreliable when used to make predictions for new data not used to build the model. Given that this concern applies to our ES meta-analytic models, we performed leave-one-out cross-validation: 1 observation was excluded at a time, the models were fitted to the remaining observations, and the fitted model was used to predict the excluded observation. This process was repeated for all observations. We assessed the predictive accuracy of the models by estimating the mean absolute percentage error. To benchmark the predictive accuracy of the models, we compared how they performed against a direct benefit transfer approach in which predictions were based solely on the average value of ES across studies in the dataset for each ES group and the tropical forest biome, thus ignoring spatial heterogeneity. We distinguished between global direct benefit transfer (that pooled all the observations per ES type) and regional direct benefit transfer (that calculated means of ES values per ES type and region).

To further assess model accuracy, we performed predicted versus observed regressions that were forced through the origin (S2 Fig).

#### Costs of operationalizing ES value internalization

Conservatively, we considered also the costs of a hypothetical internalization of the estimated value of ES. For the value of ES to be internalized by the market, payment for ES programs were assumed. These programs need to meet the opportunity costs of agriculture (see the section Economic benefits: Value of net revenues from agriculture) but also incur transaction and implementation costs. To account for transaction and implementation costs, we used as a framework the Juma project in Brazil, a program with sufficient documentation and Gold-level REDD+ status [64]. To be able to transform socioeconomic and labour cost realities different to Brazil, we expressed the preparation, administration, community support, protected area management, law enforcement, and monitoring costs per hectare and year into the person-hours required. We then used the wages in the different countries to adjust the cost categories accordingly [65] (S2 Data). These costs were deducted from the value of ES in each cell.

### Economic benefits: Value of net revenues from agriculture

To estimate the revenues generated by agricultural conversion we selected the top 10 crops in terms of area and value of production in tropical countries [66]. Since some crops appeared in both groups we were left with 18 crops. We added cattle production to the list of crops that were [66]: banana, bean, cassava, cocoa, coconut, coffee, cotton, cowpea, groundnut, maize, millet, oil palm, rice, rubber, sorghum, soybean, sugar cane, and wheat. Additionally, we accounted for the benefits of selling logged timber from deforestation prior to agricultural production. Benefits of sales of timber after land conversion were estimated by multiplying the proportion of growing stock in commercial species by the total growing stock in each region [67], then multiplying by the national export price per unit of volume [66] (final values per hectare are shown in S2 Data).

The available global crop maps corresponded to the period around the year 2000. Given this limitation we could not link specific crops to deforestation using the maps. Given this uncertainty, we estimated agricultural benefits under 2 broad scenarios. First, under scenarios A and B, we replaced converted forests with the crops already found in the country using Monte Carlo techniques that sampled crops at random from all the deforested cells in the country. Scenarios A and B are meant to represent the status quo by taking into account potentially both smallholder and industrial agricultural activities within each country. These scenarios imply that forest replacement is only possible for crops that have proven to be viable in the country in the past and that agricultural activities are more similar the closer they are in space [21]. They also invoke the requirement of know-how and infrastructure (e.g., mills and high levels of labour inputs in the case of oil palm) for each crop. We nonetheless further evaluated the plausibility of scenarios A and B following 2 approaches. First, we used a spatially explicit dataset of oil palm expansion in Southeast Asia [68,69]. If scenarios A and B were plausible, we would expect to see that new oil palm conversion occurred in the vicinity of existing oil palm plantations. We evaluated this with a model that used distance from existing plantations in 2010 as prediction of new oil palm plantations in 2014 using a generalized least-squares model. Second, we evaluated the time series of crop areas of all crops grown in the countries studied from 2000 to 2014. We evaluated whether crops not present in the countries in previous years started to be grown anew during the time series. Under our assumptions for scenarios A and B, the emergence of new crops would be unlikely over the period of study.

A second set of scenarios C and D replaced converted forest with the crop providing the highest potential rent for each cell (i.e., industrial and cash crops that involve transport costs to the market). This scenario relaxes the assumption that only national existing crops can replace forests and represents a hypothetical future scenario in which specific crop know-how and infrastructures are available in every cell and country. Scenarios A and B therefore present forests replaced by the expansion of national cropping systems with (A) and without (B) production costs (labour and fertilizer); scenarios C and D present forests replaced by the crop with the highest potential rent with (C) and without (D) production costs (labour, fertilizer, and transport costs). We derived transport costs using existing maps of travelling times to the nearest city [54], driver salaries (using agricultural wages when available and if not, manufacturing wages) [65] and fuel prices in each country for the year 2000 [70] (S2 Data shows the wages and fuel prices used). We assumed a standard truck capacity of 18 m^3^ and an average speed of 45 km/h and calculated the corresponding fuel consumption [71]. We further assumed that the truck returned empty after delivering the produce and only 1 driver was involved. Spatially explicit information on capital costs was however missing and we could not include these in the analysis. Labour costs were estimated by obtaining standard estimates of person-days per hectare and year to produce each crop in the tropics from literature review (S2 Data shows the estimated person-days and the sources for each crop) and then multiplying by the agricultural wages in each country whenever available and, if not, multiplying by the manufacturing wages [65]. Person-days estimates were coarse, as it was not possible to identify different estimates per crop and country combinations. These limitations motivated scenarios B and D that did not deduct production costs to estimate the boundaries of the uncertainty caused by production costs. We derived global maps of fertilizer costs using maps of fertilizer usage [72] and later multiplying them by the average price of fertilizer in each country from the years 2000 to 2002 [66] (S2 Data).

Conservatively, total and immediate conversion from deforestation into agricultural activities was assumed. The annual net rents from agriculture (*AR*) in each cell *i* were calculated as:

$$AR_{i}=y_{ui}p_{u}-c_{ui}$$

where *u* represents the crop assumed to occupy each deforested cell *i* under A to D scenarios; *y*_*ui*_ represents the yield of crop *u* in cell *i*; *p*_*u*_ is the farm gate price of the crop in each country per year and ton, averaged from 2000 to 2009 if available for specific countries [66] and using neighbouring countries of comparable level of development if no data were available for specific crop–country combinations (S2 Data); and *c*_*ui*_ is the production costs of crop *u* that include transport, fertilizer, and labour costs as described above depending on the scenario considered. To calculate rents from cattle we estimated average national carcass efficiencies [66,73] that were later combined with global pasture maps [47] and the number of cattle per unit of area [43]. We used 2016 international dollars to express all economic values.

### Quantifying net economic impacts

We combined our estimates of carbon emissions and ES losses with agricultural rents, assuming that they represented the marginal cost of deforestation in relation to the marginal benefits of alternative land uses [3]. We assumed that the remaining tropical forest area, after the marginal deforestation, was far from the threshold for which ES can no longer be provided, leading to a spike in value. Under this assumption, the annual economic impact, *I*, can be approximated by the following equation:

$$I=\sum_{i}^{n}\left[\left(TEV_{i}-AR_{i}\right)A_{i}\right]$$

where *TEV* is the value of the externalities because of carbon emissions (annualized using a discount rate of 5% and a 100 years’ time horizon) and annual ES losses and *AR* represents net benefits from logging (annualized using a similar discount rate and time period) plus annual rents from agriculture of converting the area deforested *A* in cell *i*.

### Analysis of uncertainty

Our analysis involved the combination of several spatial datasets and analyses with inherent uncertainty on their own. The combination of individual sources of uncertainty may lead to a greater uncertainty in the estimates than individual uncertainties alone. There was thus a need to explicitly model each source of uncertainty and to propagate this uncertainty to estimate what was the combined uncertainty generated by the analyses. As noted, we used several scenarios and sampled at random from agricultural fields to account for uncertainty in production costs and the distribution of crops replacing forests. We further dealt with uncertainty using bootstrapping methods in the case of the meta-analytic models of ES. We then employed uncertainty distributions to take into account uncertainty in the crop prices, the carbon prices, and the deforestation maps. Monte Carlo simulation methods were then used to sample 200 times from each source of uncertainty to generate uncertainty distributions of potential outcomes for each cell of the map.

Specifically, we bootstrapped each of the 3 selected ES models to evaluate their predictive uncertainty using the function bootMer from the lme4 package [63]. We carried out 500 bootstraps that involved resampling the dataset, fitting the model again and producing predictions for each type of ES in the map at a 0.1° resolution across the global tropical forest biome. As a result, a distribution (*n* = 500) of ES lost annually in each cell in the map was obtained (i.e., 500 different maps of total ES values). Among these 500 maps, 1 map was chosen at random each time the model was run.

The uncertainty in the deforestation maps was modelled by modifying the original dataset stochastically to reflect 87% and 99.7% accuracy in the classification of forest loss and no loss in the tropics [15] (i.e., each cell classified as forest lost had a probability of 0.87 of remaining as forest loss and 0.13 of being changed to not forest loss in each run). Maps of forest loss were modified stochastically according to these probabilities every time the model was run.

To account for the uncertainty in market prices of carbon, we considered the daily time series of prices from August of 2011 to September of 2016. The time series was sampled at random every time the model was run, and the sampled value used as the market price of carbon. For the social price of carbon, we considered 1,000 observations per each of the 5 modelling scenarios considered and sampled at random among these 5,000 values to select the social price of carbon each time the model was run. For agricultural prices, we estimated the standard deviation of the prices for each crop–country combination from 2000 to 2009. We then constructed normal distributions using the mean and standard deviations of the observed prices. The normal distributions were sampled each time the model was run. The final combination of uncertainty sampling using Monte Carlo methods led to a distribution of model outcomes. 2.5th and 97th percentiles of the distributions of outputs generated were estimated and used to generate uncertainty ranges of the results.

## Supporting information

S1 Fig Final meta-analytic model resulting from conditional averaging of the top 3 models. “Provisioning” and “Regulating” are levels of ecosystem services types compared against cultural services. “Revealed preference” and “Stated preference” are levels of valuation methods and compared against cost-based valuation methods.
(TIF)

S2 Fig Predicted versus observed linear regressions for the top 3 models obtained through information theory. Predictions were generated based on a leave-one-out cross-validation procedure. β denotes the slope of the regression and R^2^ represents the proportion of the variance explained by the regression.
(TIF)

S3 Fig The economic implications of deforestation in tropical forests from 2000 to 2012. Comparison of carbon emissions assessed at market prices plus loss of ES values (TEVm) minus gains of agricultural rents under scenario A (AR1). Values at the 2.5th percentile of the simulations are shown.
(TIF)

S4 Fig The economic implications of deforestation in tropical forests from 2000 to 2012. Comparison of carbon emissions assessed at market prices plus loss of ES values (TEVm) minus gains of agricultural rents under scenario A (AR1). Values at the 97.5th percentile of the simulations are shown.
(TIF)

S5 Fig Spatial distribution of the value of CO_2_ emissions from deforestation from 2000–2012 under market prices. Median values of the simulations are shown.
(TIF)

S6 Fig Spatial distribution of the value of CO_2_ emissions from deforestation from 2000–2012 under social prices. Median values of the simulations are shown.
(TIF)

S7 Fig Annual per-country net benefits and losses of converting tropical forests to agriculture for the years 2000–2012 compared to total ecosystem services losses. This figure shows the result of deducting agricultural rents (AR) from the crops replacing forests under 4 different scenarios (A, B, C, and D with corresponding agricultural rents AR1, AR2, AR3, and AR4) to the total ecosystem value based on the social price of carbon (TEVs). Error bars indicate the 2.5th and 97.5th percentiles of the uncertainty distribution of outcomes.
(TIF)

S8 Fig Annual per-country net benefits and losses of converting tropical forests to agriculture for the years 2000–2012 compared to carbon emissions. This figure shows the result of deducting agricultural rents (AR) from the crops replacing forests under 4 different scenarios (A, B, C, and D with corresponding agricultural rents AR1, AR2, AR3, and AR4) to the total value of CO_2_ emissions under market prices (CO_2_m). Error bars indicate the 2.5th and 97.5th percentiles of the uncertainty distribution of outcomes.
(TIF)

S9 Fig Annual per-country net benefits and losses of converting tropical forests to agriculture for the years 2000–2012 compared to carbon emissions. This figure shows the result of deducting agricultural rents (AR) from the crops replacing forests under 4 different scenarios (A, B, C, and D with corresponding agricultural rents AR1, AR2, AR3, and AR4) to the total value of CO_2_ emissions under social prices (CO_2_s). Error bars indicate the 2.5th and 97.5th percentiles of the uncertainty distribution of outcomes.
(TIF)

S10 Fig The economic implications of deforestation in tropical forests from 2000 to 2012. Comparison of carbon emissions assessed at social prices minus gains of agricultural rents under scenario A (AR1). The median values of the simulations are shown.
(TIF)

S11 Fig The economic implications of deforestation in tropical forests from 2000 to 2012. Comparison of carbon emissions assessed at social prices minus gains of agricultural rents under scenario B (AR2). The median values of the simulations are shown.
(TIF)

S12 Fig The economic implications of deforestation in tropical forests from 2000 to 2012. Comparison of carbon emissions assessed at market prices plus loss of ES values (TEVm) minus gains of agricultural rents under scenario B (AR2). Values at the 2.5th percentile of the simulations are shown.
(TIF)

S13 Fig The economic implications of deforestation in tropical forests from 2000 to 2012. Comparison of carbon emissions assessed at market prices plus loss of ES values (TEVm) minus gains of agricultural rents under scenario B (AR2). Median values of the simulations are shown.
(TIF)

S14 Fig The economic implications of deforestation in tropical forests from 2000 to 2012. Comparison of carbon emissions assessed at market prices plus loss of ES values (TEVm) minus gains of agricultural rents under scenario B (AR2). Values at the 97.5th percentile of the simulations are shown.
(TIF)

S15 Fig The economic implications of deforestation in tropical forests from 2000 to 2012. Comparison of carbon emissions assessed at market prices plus loss of ES values (TEVm) minus gains of agricultural rents under scenario C (AR3). Values at the 2.5th percentile of the simulations are shown.
(TIF)

S16 Fig The economic implications of deforestation in tropical forests from 2000 to 2012. Comparison of carbon emissions assessed at market prices plus loss of ES values (TEVm) minus gains of agricultural rents under scenario C (AR3). Median values of the simulations are shown.
(TIF)

S17 Fig The economic implications of deforestation in tropical forests from 2000 to 2012. Comparison of carbon emissions assessed at market prices plus loss of ES values (TEVm) minus gains of agricultural rents under scenario C (AR3). Values at the 97.5th percentile of the simulations are shown.
(TIF)

S18 Fig Oil palm expansion in insular Southeast Asia from 2010 (A) to 2014 (B). New oil palm conversions are typically in the vicinity of existing plantations. A generalized least-squares model of new conversion as a function of distance from plantation in 2010 presented a coefficient of –0.06 (*p*-value < 0.01), showing that occurrence of new conversion decreased with distance from existing plantations. Data from Miettienen et al. [74] were used to build the map.
(TIF)

S19 Fig Semivariograms of top 3 ES meta-analytic models with highest support. Nonincreasing semivariance with distance denotes no problems of spatial autocorrelation in the residuals of the models. Top left, top right, bottom left: first, second, and third most supported models.
(TIF)

S1 Table Composition of the top meta-analytic models and their predictive errors. These models presented less than 2 small sample-size corrected Akaike information criterion (AICc) units of difference from the model with lowest AICc (AIC_0_). The predictive error (PE) was measured as the mean absolute predictive error. The comparisons with direct benefit transfer methods indicate changes in PE showing that the meta-analytic model causes reductions of error.
(XLSX)

S2 Table Representativeness of the TEEB database of tropical forests for the variables used in the meta-analytic model. PA: protected area status IUCN categories. perc.: percentile.
(XLSX)

S3 Table Country-crop combinations that emerged (out of 2,903 combinations) as new during the period from 2000 to 2014 in FAOSTAT [66] for all the countries considered in the study. Area information was either missing or 0.
(XLSX)

S1 Data Data used for the construction of the ecosystem services meta-analytic models. (XLSX)

S2 Data Economic parameters and inputs used in the analysis. Economic values are expressed in I$ of 2016. Person-days per crop include the studies used as source. Every crop presents farm gate prices from FAOSTAT except rubber, for which international prices were employed because of data paucity. Prices for countries not growing the commodity were set as 0.
(XLSX)

## Abbreviations

AR: agricultural rent
ES: ecosystem services
GDP: gross domestic product
GIS: geographic information systems
REDD+: Reducing Emissions from Deforestation and forest Degradation
TEEB: The Economics of Ecosystems and Biodiversity
TEV: total ecosystem service value
UR: uncertainty range
