# Supplementary figures and images for: Global economic trade-offs between wild nature and tropical agriculture
Source: PLoS Biol. 2017 Jul 21;15(7):e2001657. doi: 10.1371/journal.pbio.2001657 (PMC5521733; doi:10.1371/journal.pbio.2001657)

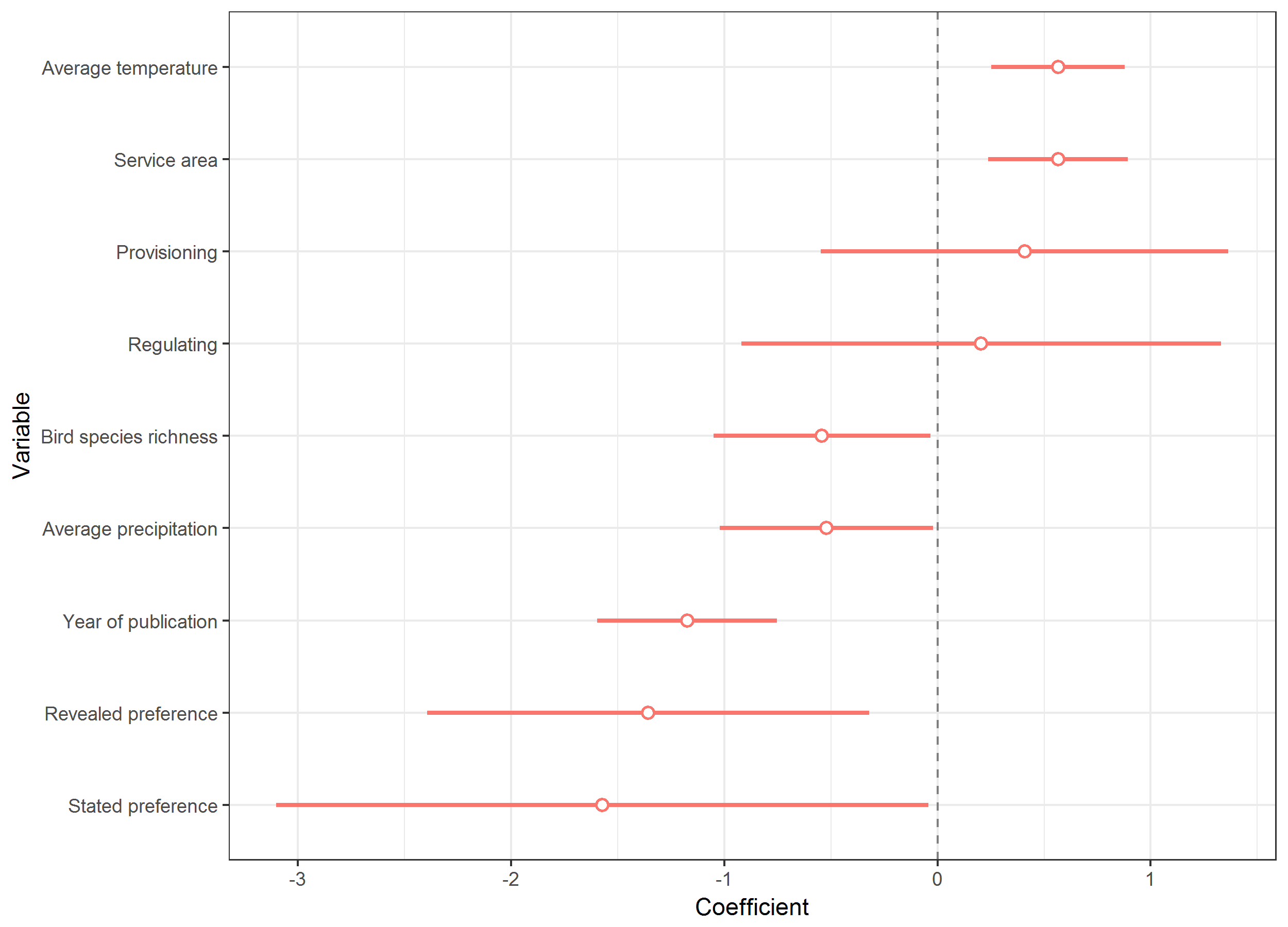

Supplement: S1 Fig — “Provisioning” and “Regulating” are levels of ecosystem services types compared against cultural services. “Revealed preference” and “Stated preference” are levels of valuation methods and compared against cost-based valuation methods. (TIF) [file pbio.2001657.s001.tiff]

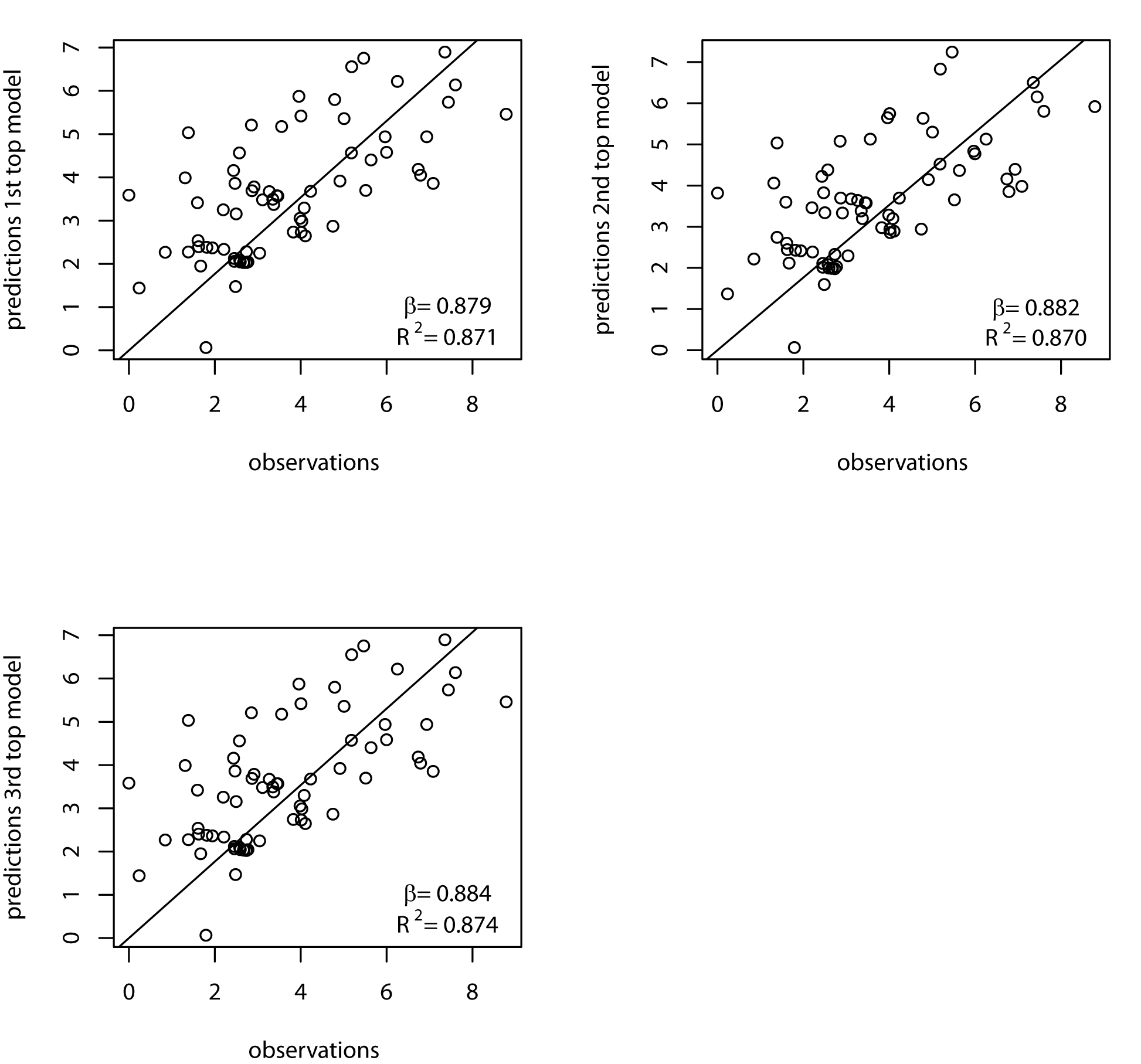

Supplement: S2 Fig — Predictions were generated based on a leave-one-out cross-validation procedure. β denotes the slope of the regression and R2 represents the proportion of the variance explained by the regression. (TIF) [file pbio.2001657.s002.tif]

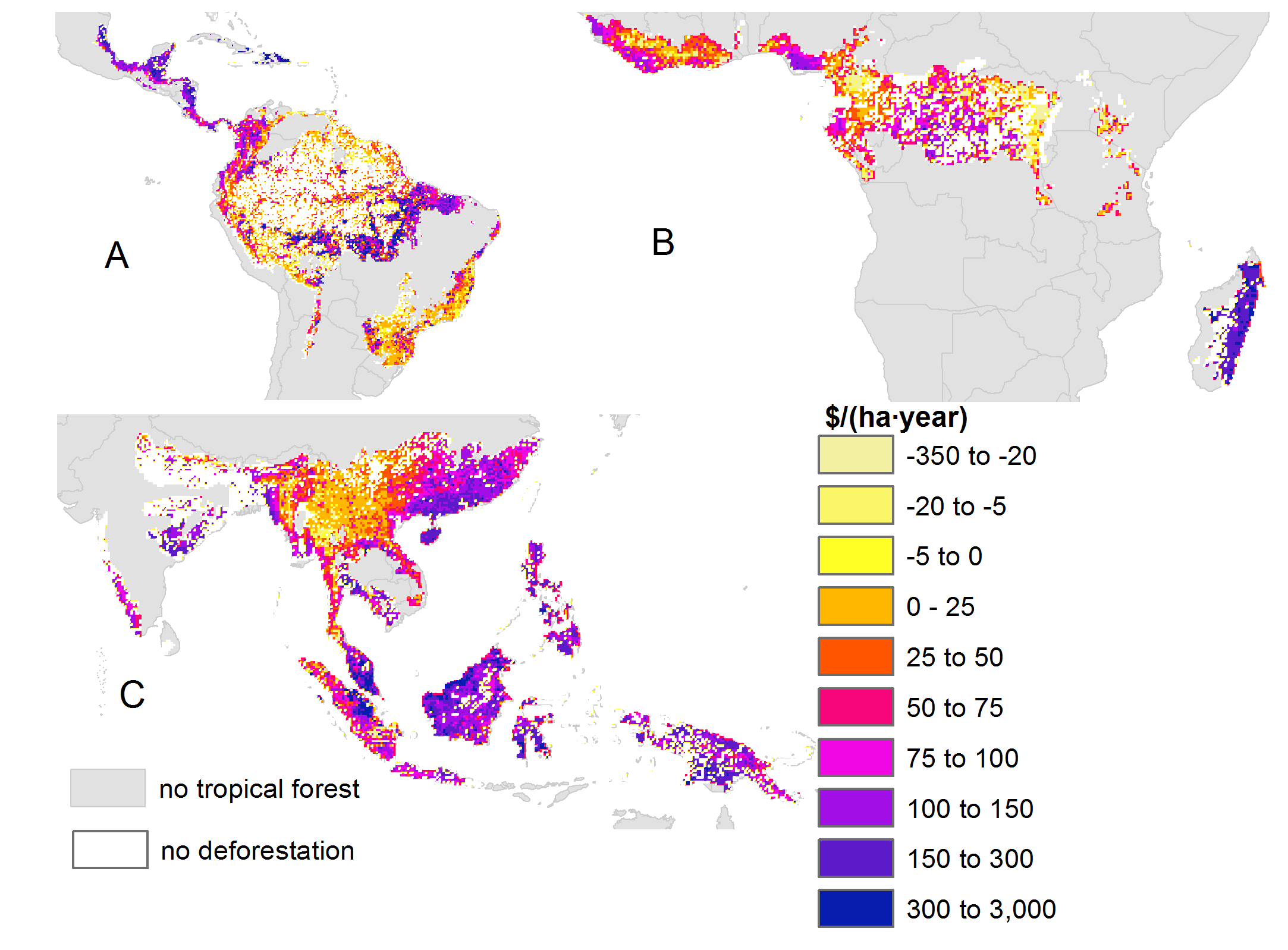

Supplement: S3 Fig — Comparison of carbon emissions assessed at market prices plus loss of ES values (TEVm) minus gains of agricultural rents under scenario A (AR1). Values at the 2.5th percentile of the simulations are shown. (TIF) [file pbio.2001657.s003.tif]

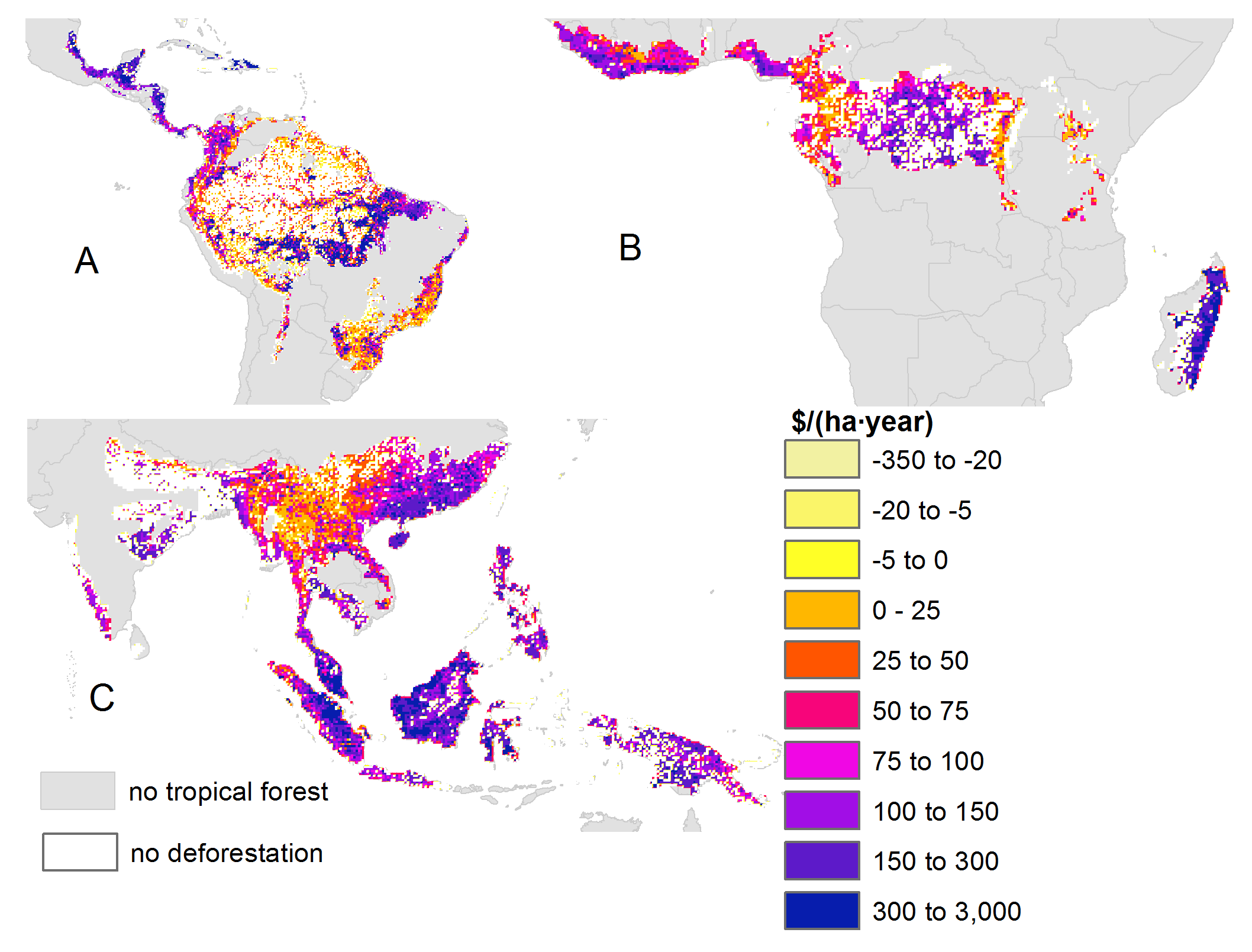

Supplement: S4 Fig — Comparison of carbon emissions assessed at market prices plus loss of ES values (TEVm) minus gains of agricultural rents under scenario A (AR1). Values at the 97.5th percentile of the simulations are shown. (TIF) [file pbio.2001657.s004.tif]

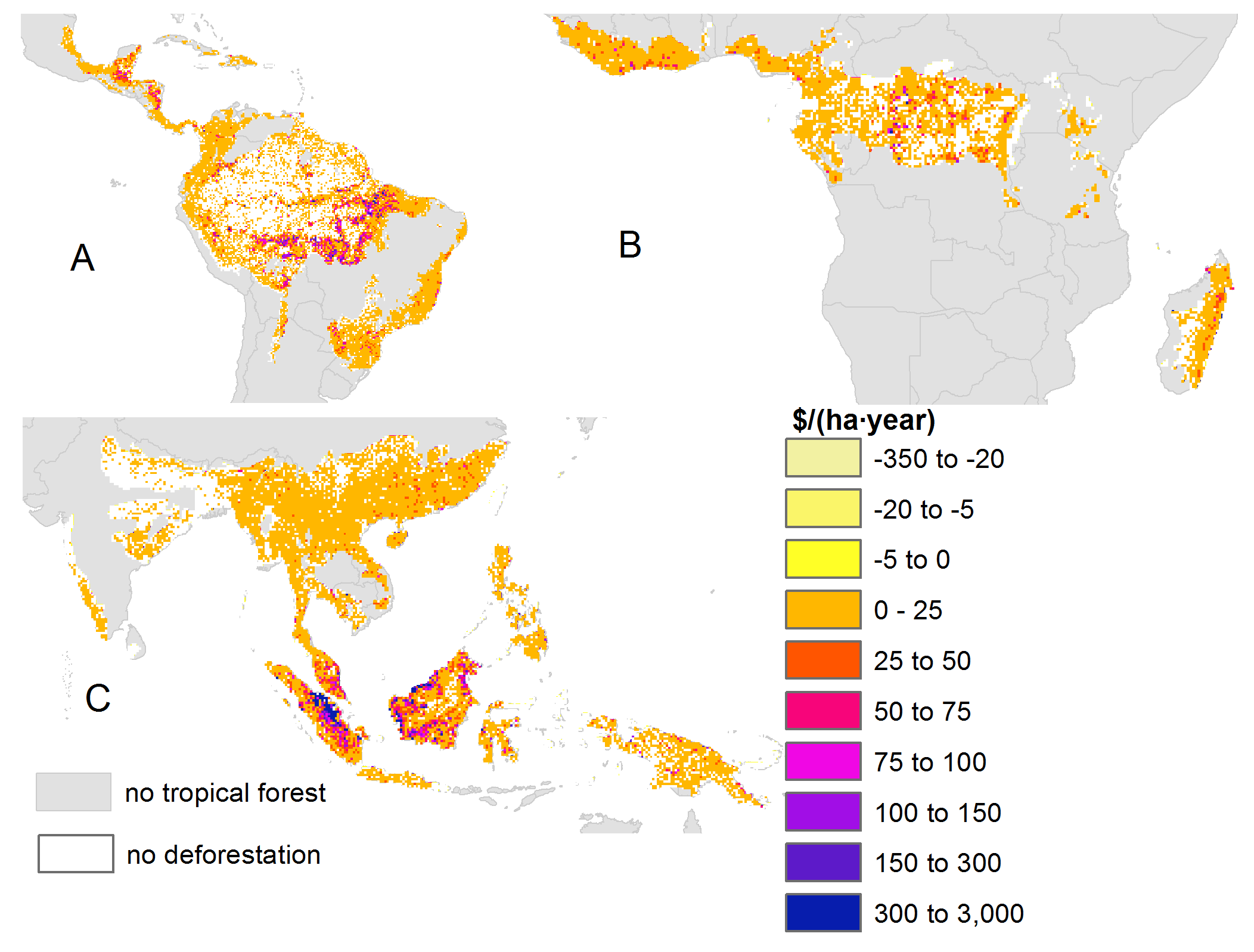

Supplement: S5 Fig — Median values of the simulations are shown. (TIF) [file pbio.2001657.s005.tif]

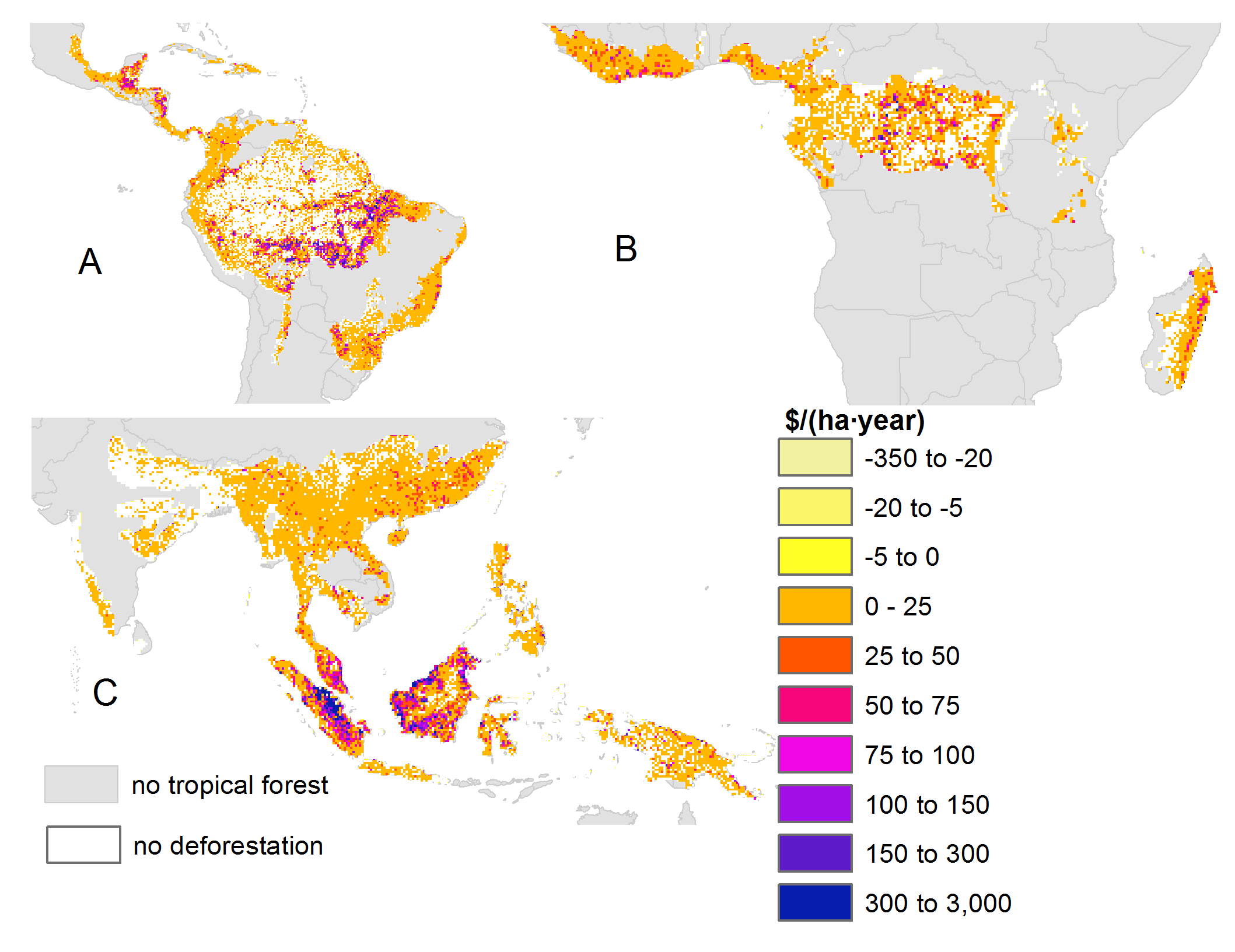

Supplement: S6 Fig — Median values of the simulations are shown. (TIF) [file pbio.2001657.s006.tif]

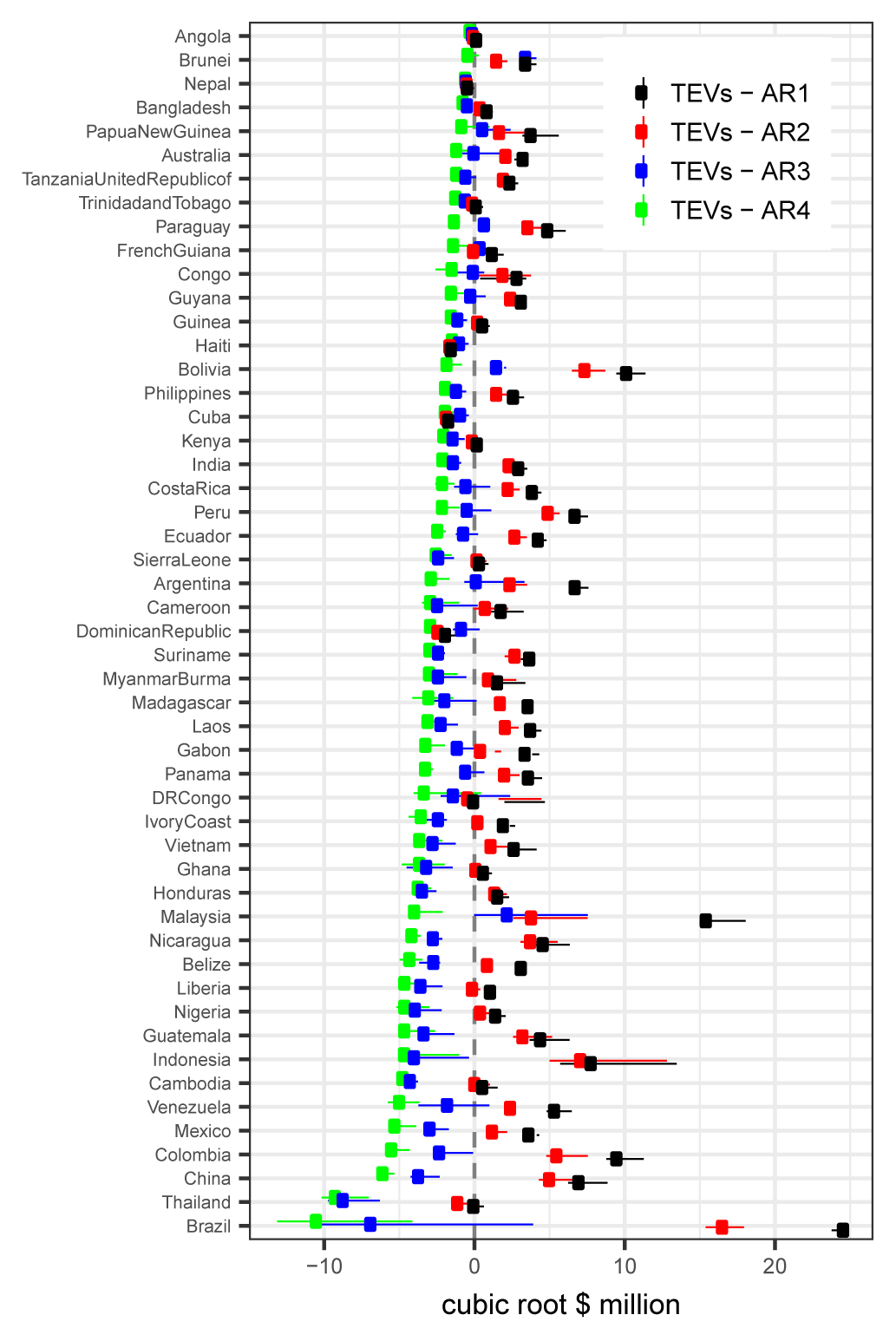

Supplement: S7 Fig — This figure shows the result of deducting agricultural rents (AR) from the crops replacing forests under 4 different scenarios (A, B, C, and D with corresponding agricultural rents AR1, AR2, AR3, and AR4) to the total ecosystem value based on the social price of carbon (TEVs). Error bars indicate the 2.5th and 97.5th percentiles of the uncertainty distribution of outcomes. (TIF) [file pbio.2001657.s007.tif]

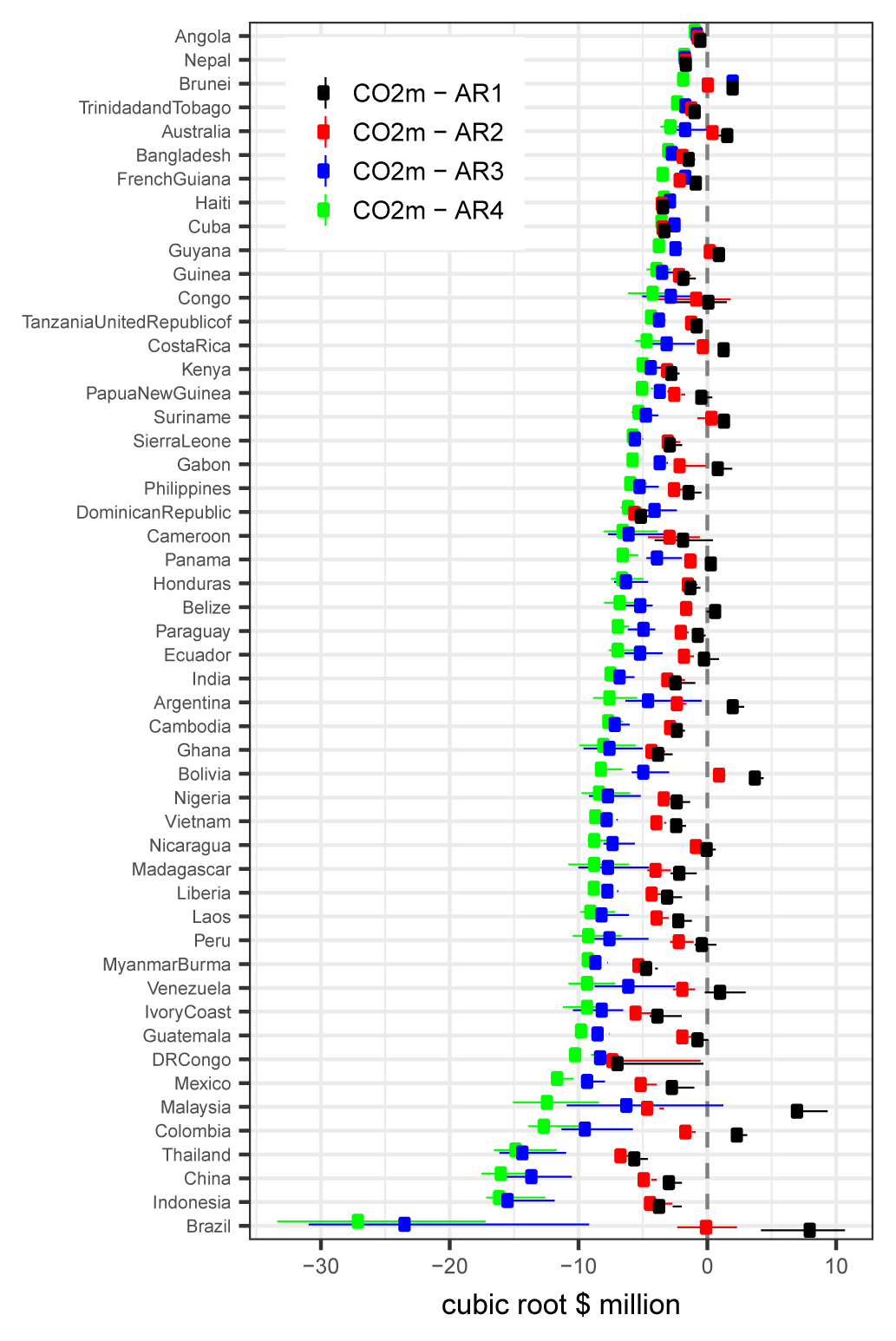

Supplement: S8 Fig — This figure shows the result of deducting agricultural rents (AR) from the crops replacing forests under 4 different scenarios (A, B, C, and D with corresponding agricultural rents AR1, AR2, AR3, and AR4) to the total value of CO2 emissions under market prices (CO2m). Error bars indicate the 2.5th and 97.5th percentiles of the uncertainty distribution of outcomes. (TIF) [file pbio.2001657.s008.tif]

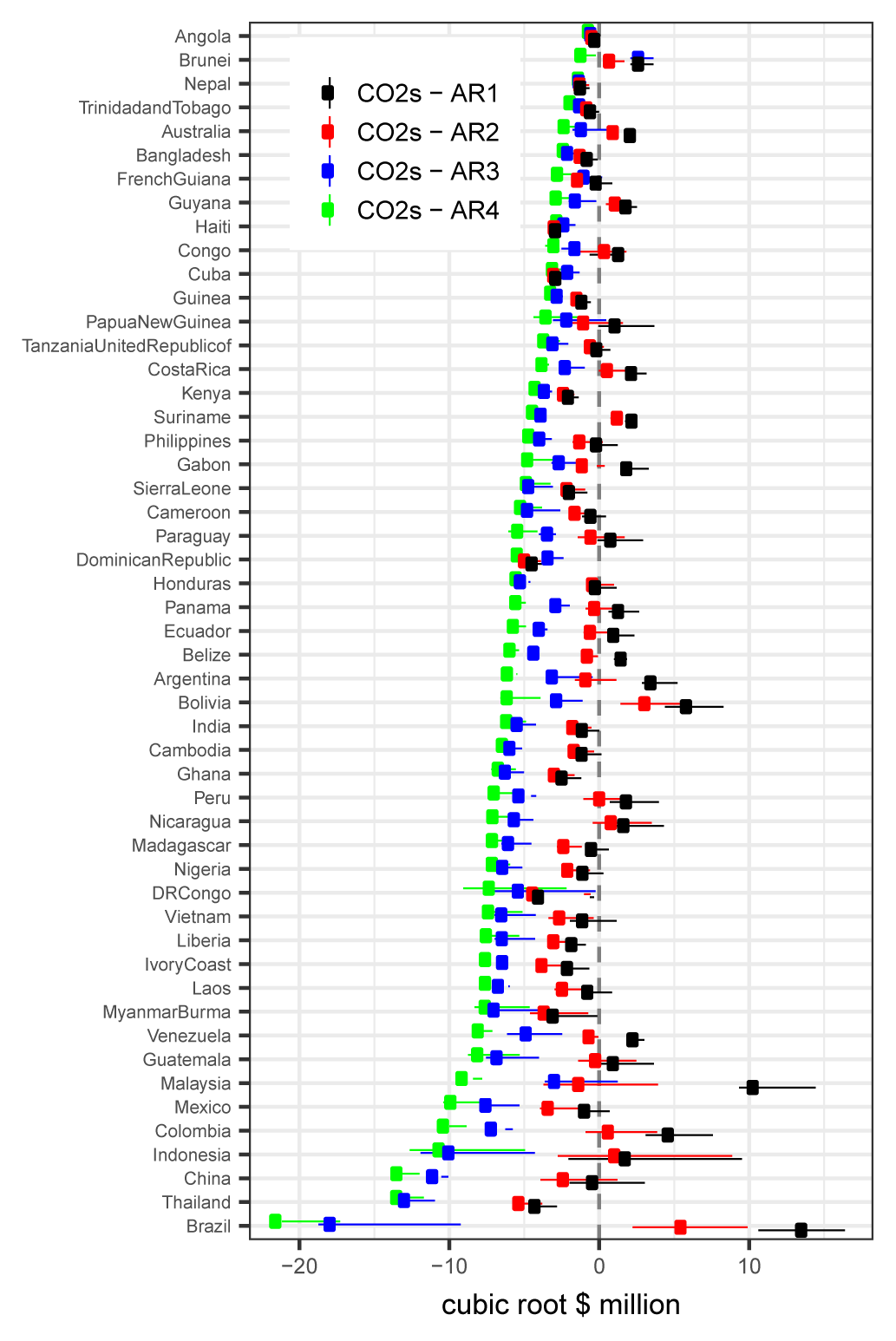

Supplement: S9 Fig — This figure shows the result of deducting agricultural rents (AR) from the crops replacing forests under 4 different scenarios (A, B, C, and D with corresponding agricultural rents AR1, AR2, AR3, and AR4) to the total value of CO2 emissions under social prices (CO2s). Error bars indicate the 2.5th and 97.5th percentiles of the uncertainty distribution of outcomes. (TIF) [file pbio.2001657.s009.tif]

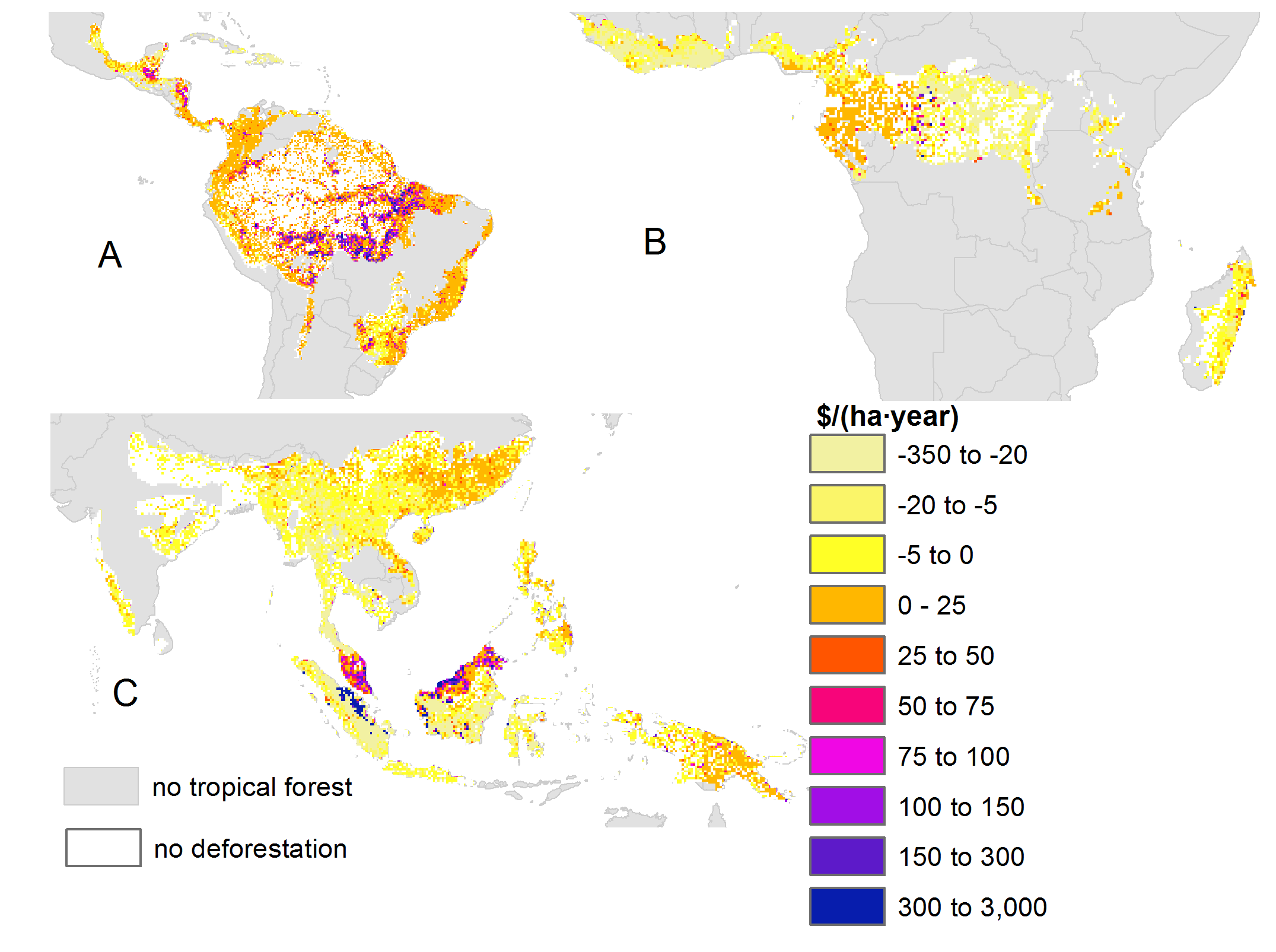

Supplement: S10 Fig — Comparison of carbon emissions assessed at social prices minus gains of agricultural rents under scenario A (AR1). The median values of the simulations are shown. (TIF) [file pbio.2001657.s010.tif]

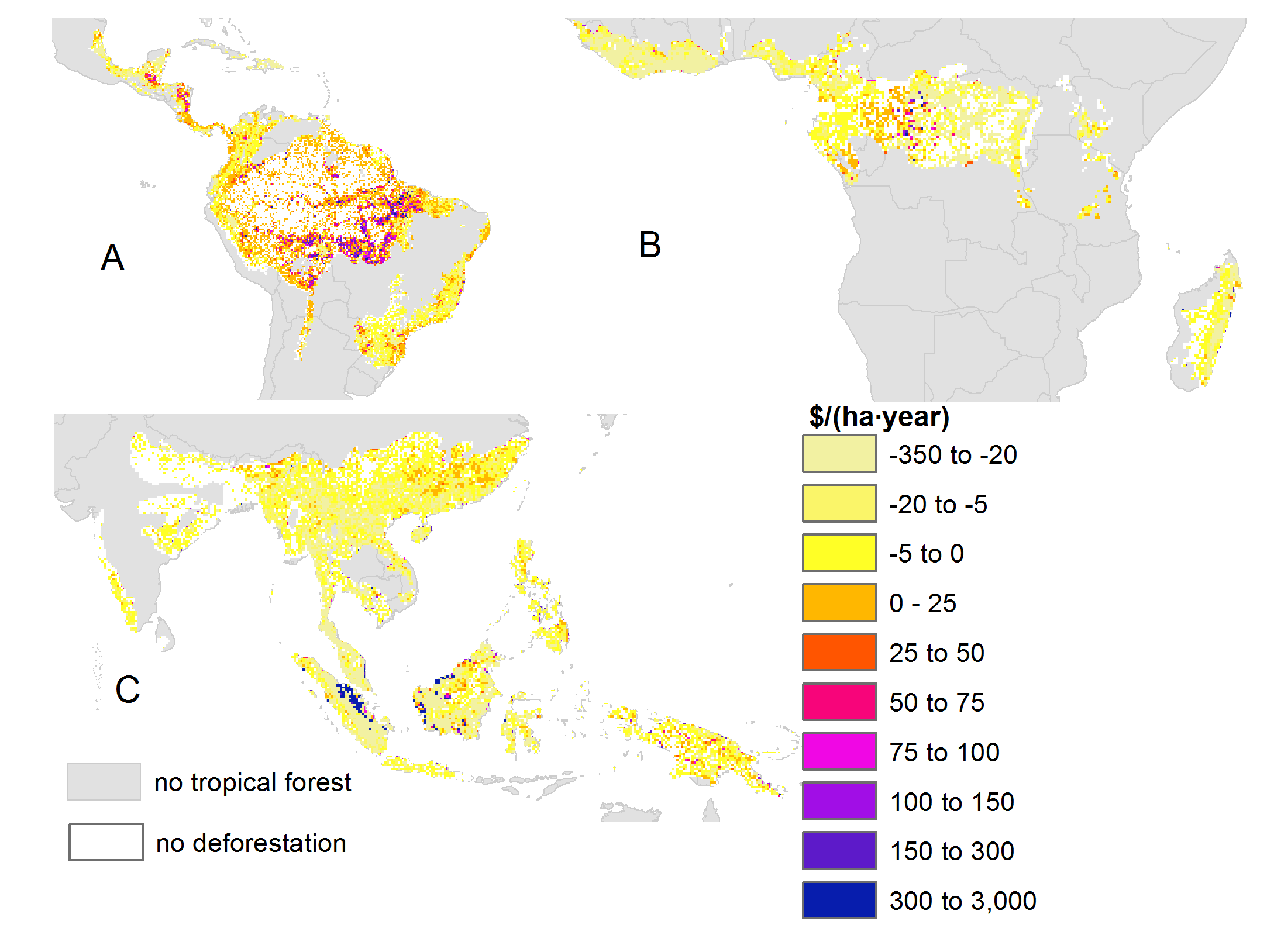

Supplement: S11 Fig — Comparison of carbon emissions assessed at social prices minus gains of agricultural rents under scenario B (AR2). The median values of the simulations are shown. (TIF) [file pbio.2001657.s011.tif]

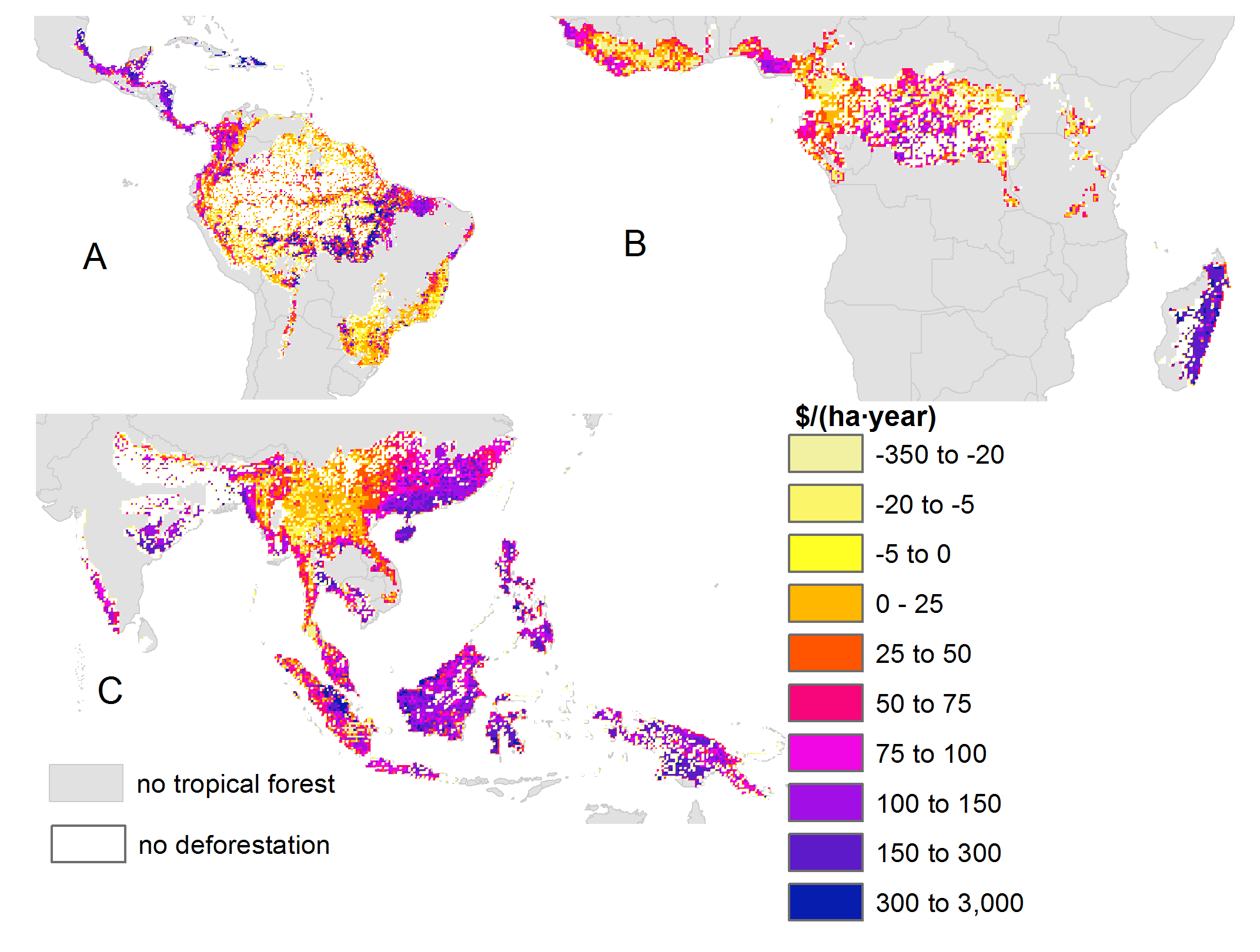

Supplement: S12 Fig — Comparison of carbon emissions assessed at market prices plus loss of ES values (TEVm) minus gains of agricultural rents under scenario B (AR2). Values at the 2.5th percentile of the simulations are shown. (TIF) [file pbio.2001657.s012.tif]

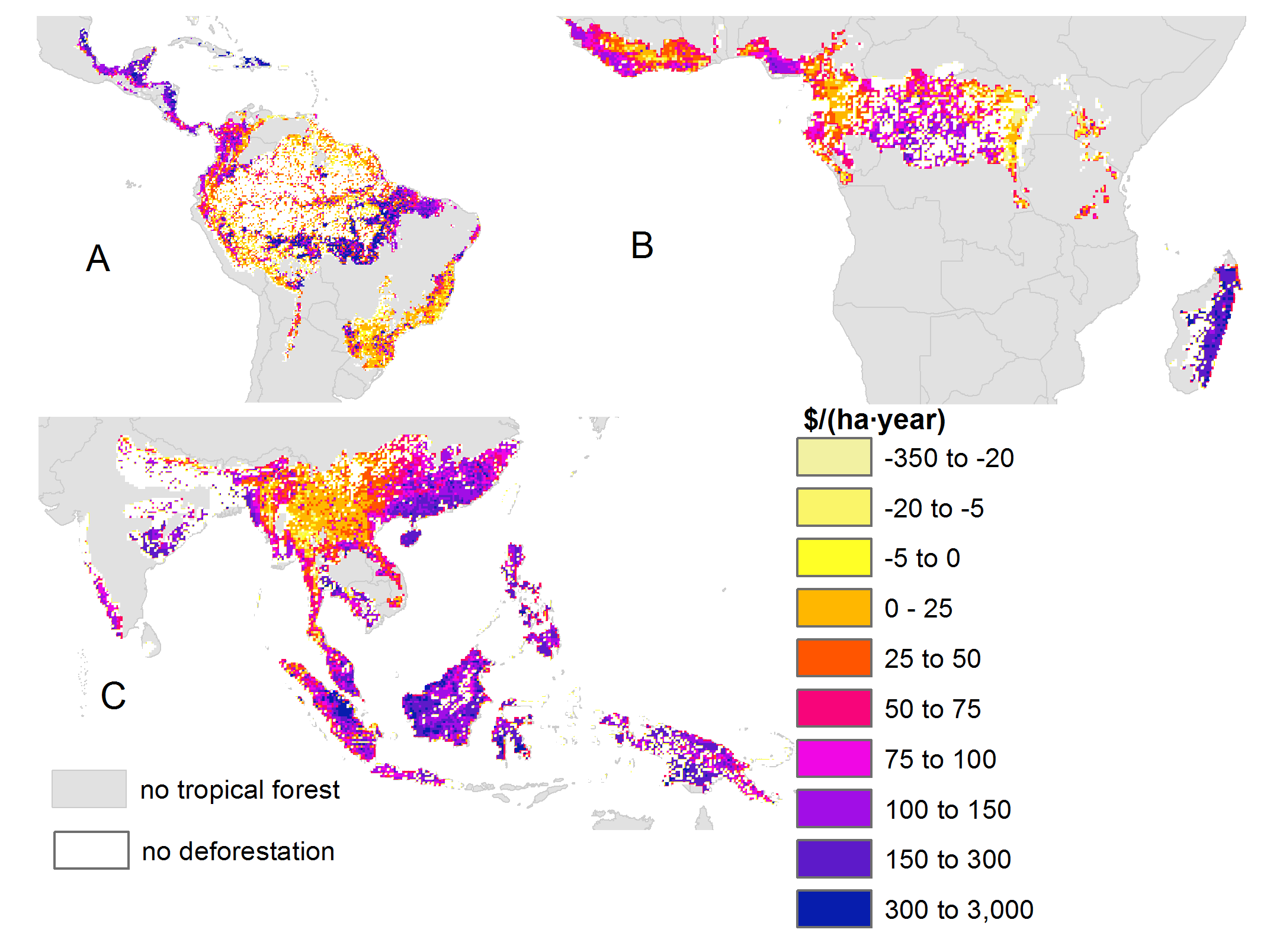

Supplement: S13 Fig — Comparison of carbon emissions assessed at market prices plus loss of ES values (TEVm) minus gains of agricultural rents under scenario B (AR2). Median values of the simulations are shown. (TIF) [file pbio.2001657.s013.tif]

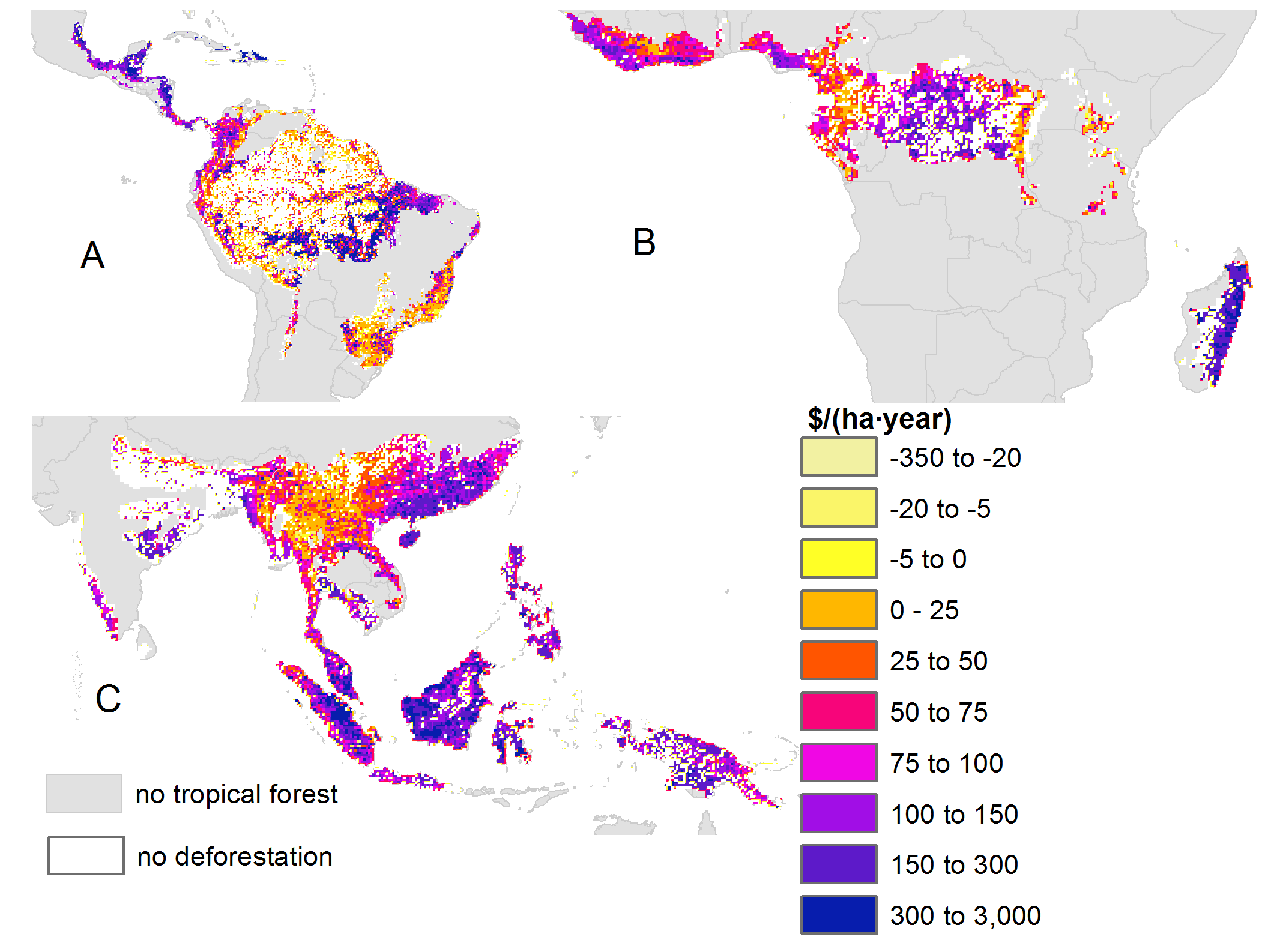

Supplement: S14 Fig — Comparison of carbon emissions assessed at market prices plus loss of ES values (TEVm) minus gains of agricultural rents under scenario B (AR2). Values at the 97.5th percentile of the simulations are shown. (TIF) [file pbio.2001657.s014.tif]

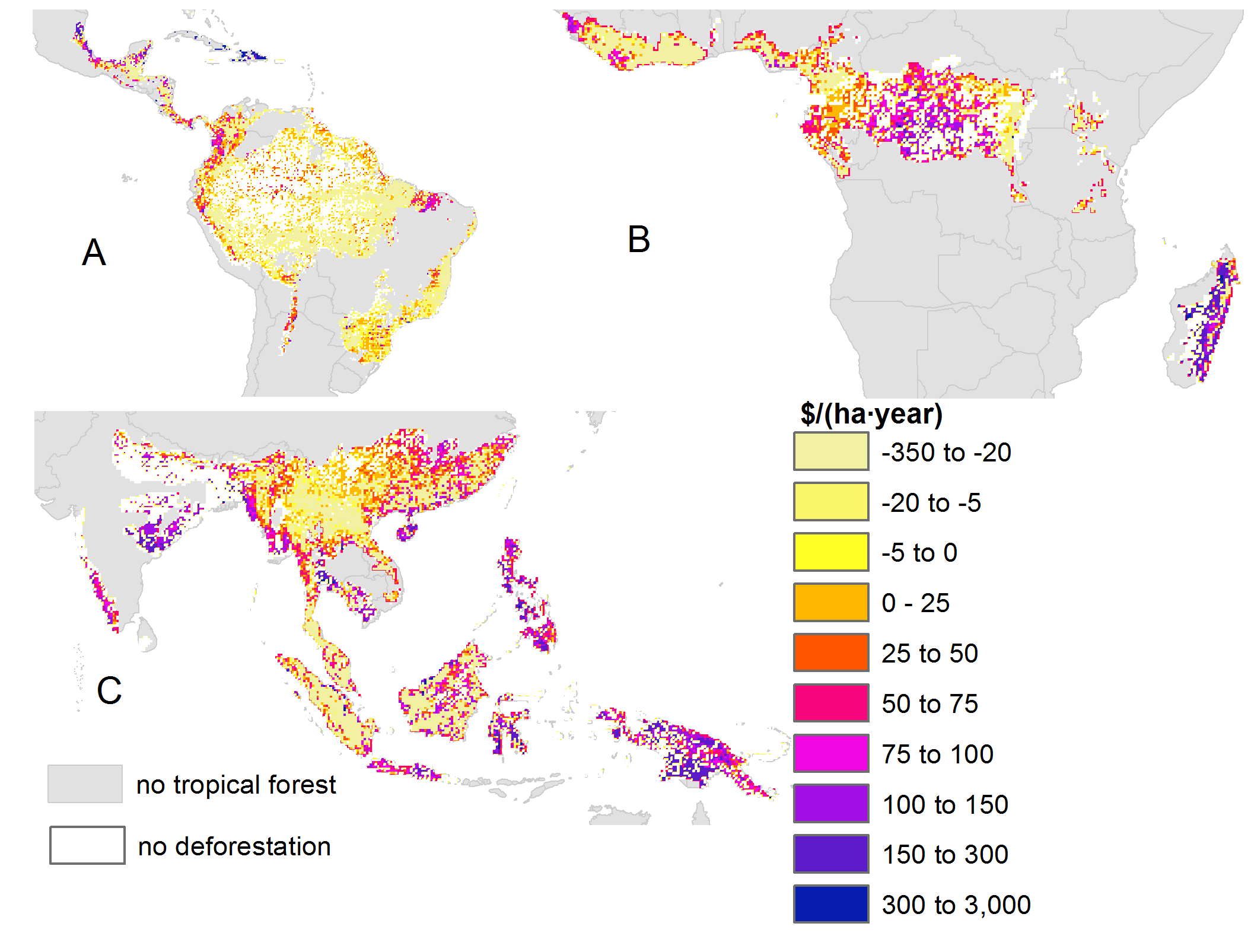

Supplement: S15 Fig — Comparison of carbon emissions assessed at market prices plus loss of ES values (TEVm) minus gains of agricultural rents under scenario C (AR3). Values at the 2.5th percentile of the simulations are shown. (TIF) [file pbio.2001657.s015.tif]

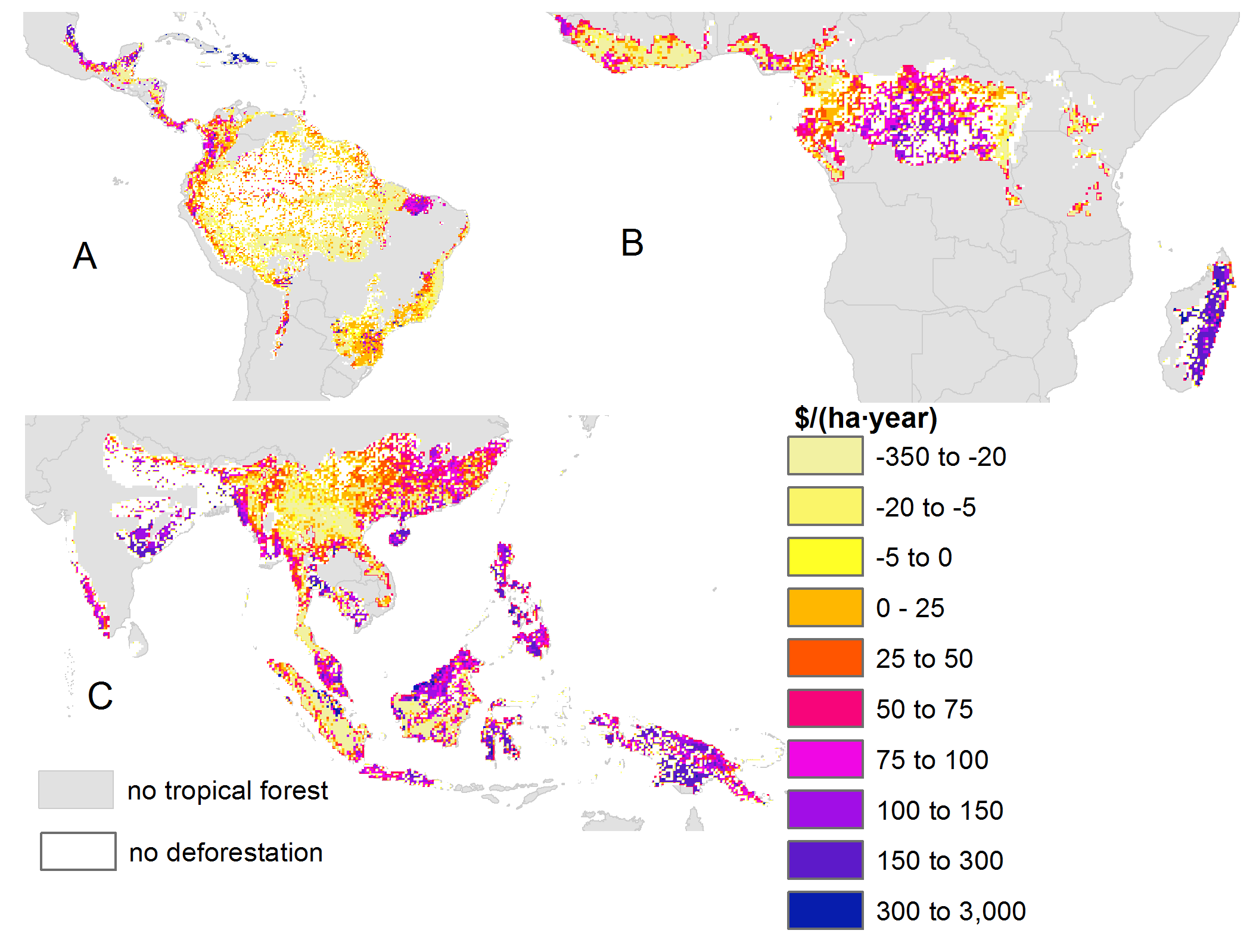

Supplement: S16 Fig — Comparison of carbon emissions assessed at market prices plus loss of ES values (TEVm) minus gains of agricultural rents under scenario C (AR3). Median values of the simulations are shown. (TIF) [file pbio.2001657.s016.tif]

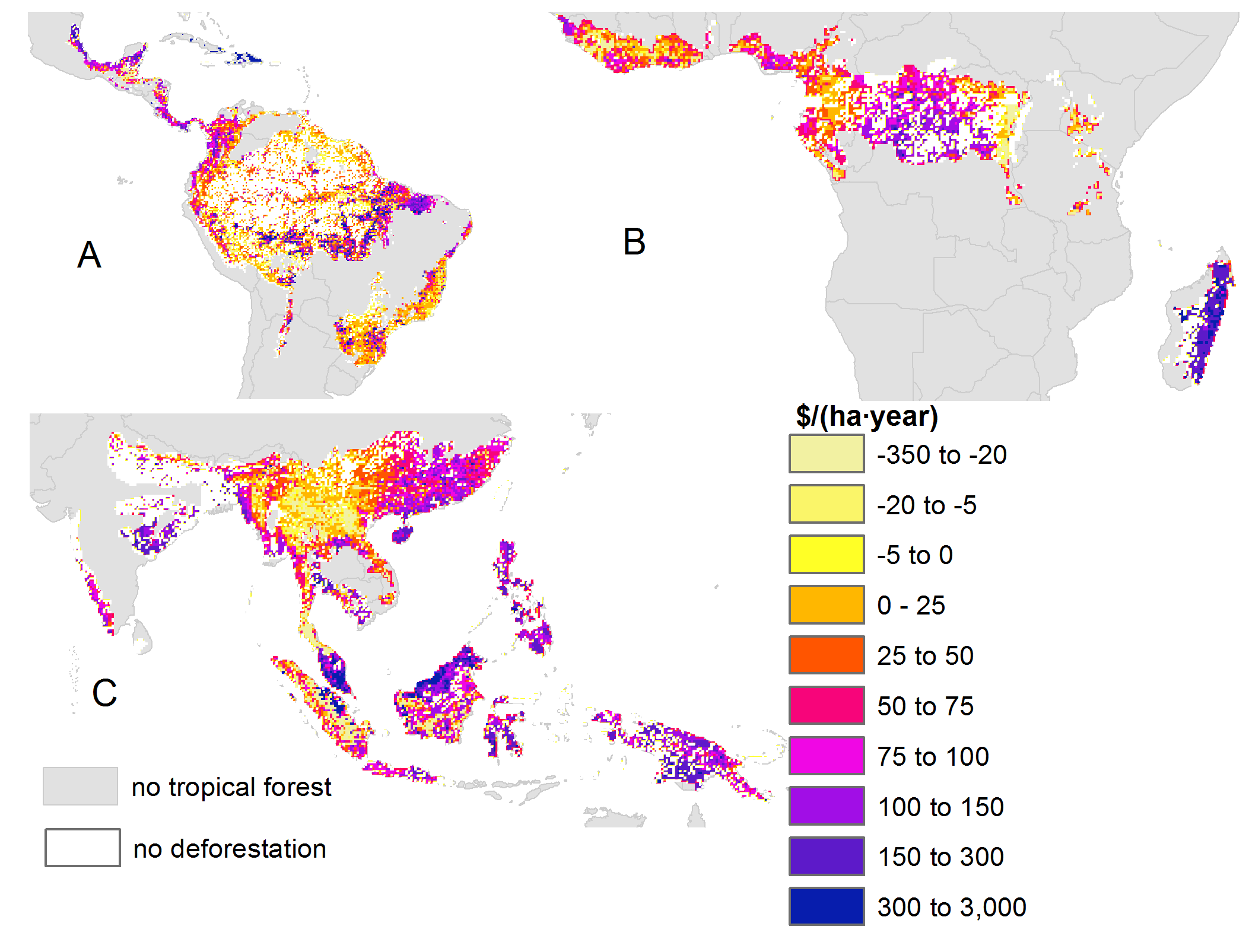

Supplement: S17 Fig — Comparison of carbon emissions assessed at market prices plus loss of ES values (TEVm) minus gains of agricultural rents under scenario C (AR3). Values at the 97.5th percentile of the simulations are shown. (TIF) [file pbio.2001657.s017.tif]

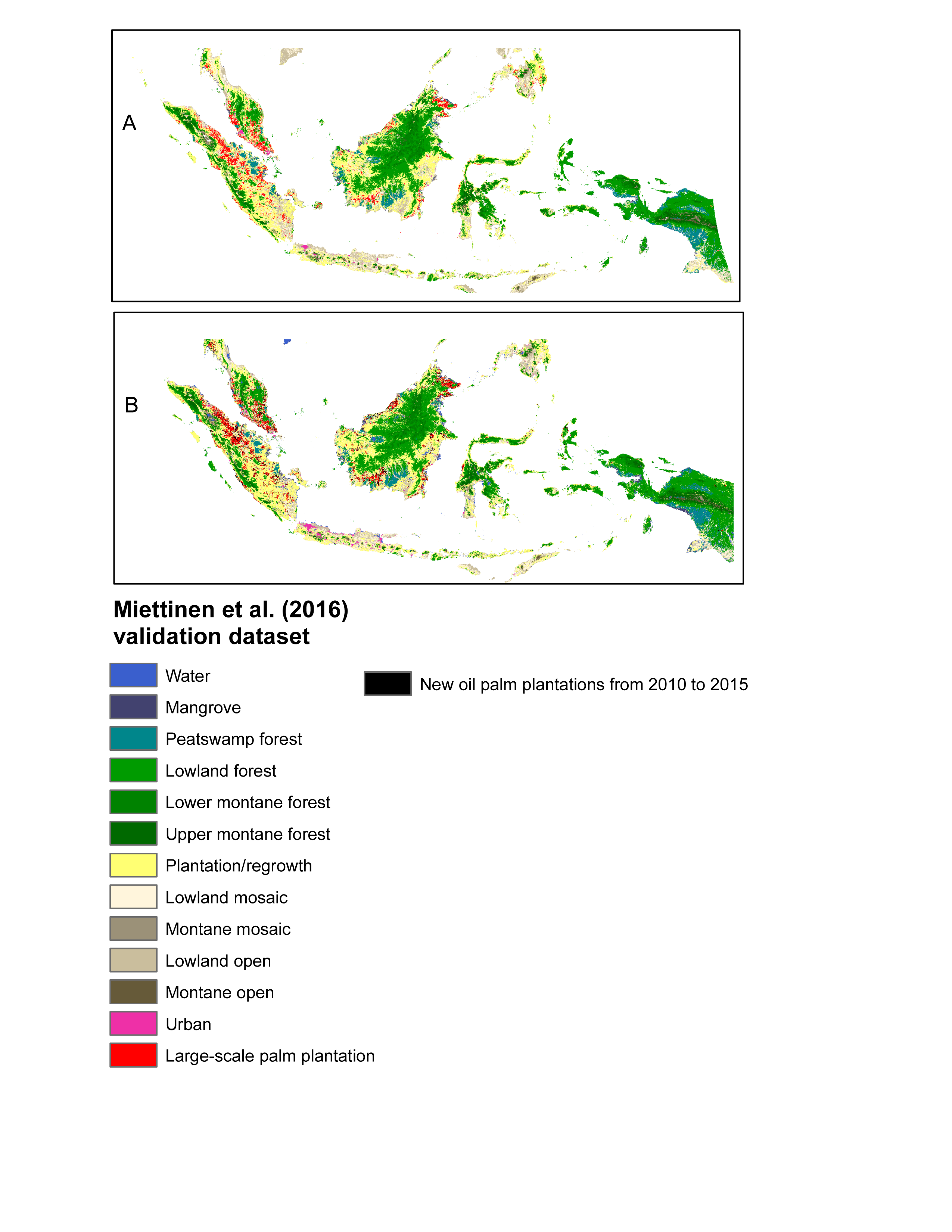

Supplement: S18 Fig — New oil palm conversions are typically in the vicinity of existing plantations. A generalized least-squares model of new conversion as a function of distance from plantation in 2010 presented a coefficient of –0.06 (p-value < 0.01), showing that occurrence of new conversion decreased with distance from existing plantations. Data from Miettienen et al. [74] were used to build the map. (TIF) [file pbio.2001657.s018.tif]

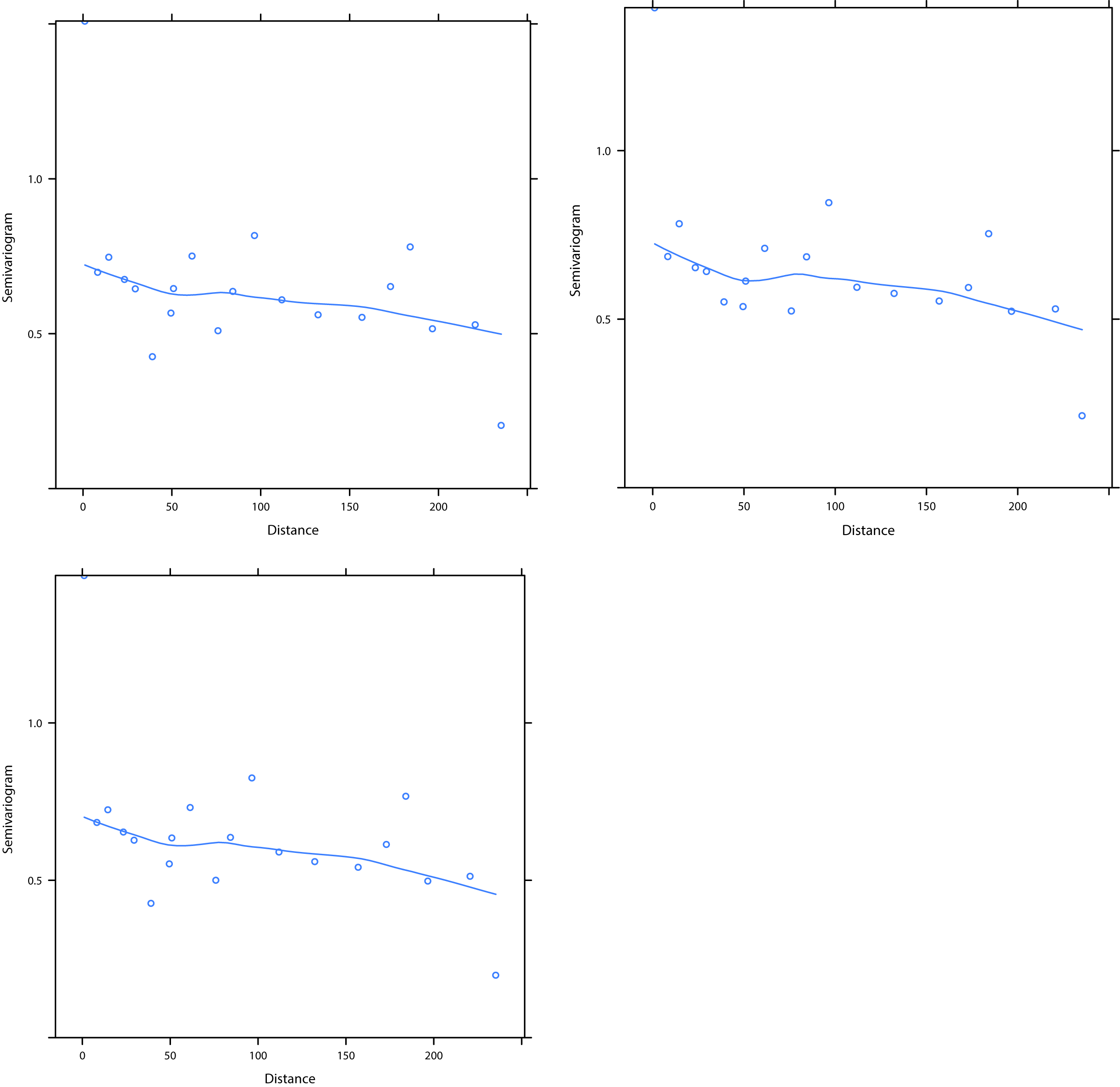

Supplement: S19 Fig — Nonincreasing semivariance with distance denotes no problems of spatial autocorrelation in the residuals of the models. Top left, top right, bottom left: first, second, and third most supported models. (TIF) [file pbio.2001657.s019.tif]
